# The Zinc Finger Protein Mig1 Regulates Mitochondrial Function and Azole Drug Susceptibility in the Pathogenic Fungus *Cryptococcus neoformans*

**DOI:** 10.1128/mSphere.00080-15

**Published:** 2016-01-13

**Authors:** Mélissa Caza, Guanggan Hu, Michael Price, John R. Perfect, James W. Kronstad

**Affiliations:** a Department of Microbiology and Immunology, Michael Smith Laboratories, University of British Columbia, Vancouver, Canada; b Department of Medicine and Molecular Genetics and Microbiology, Duke University Medical Center, Duke University, Durham, North Carolina, USA; Carnegie Mellon University

**Keywords:** carbon metabolism, cell wall, macrophages, regulation, TOR kinase

## Abstract

The opportunistic pathogen *Cryptococcus neoformans* causes fungal meningoencephalitis in immunocompromised individuals. In previous studies, we found that the Hap complex in this pathogen represses genes encoding mitochondrial respiratory functions and tricarboxylic acid (TCA) cycle components under low-iron conditions. The orthologous Hap2/3/4/5 complex in *Saccharomyces cerevisiae* exerts a regulatory influence on mitochondrial functions, and Hap4 is subject to glucose repression via the carbon catabolite repressor Mig1. In this study, we explored the regulatory link between a candidate ortholog of the Mig1 protein and the HapX component of the Hap complex in *C. neoformans*. This analysis revealed repression of *MIG1* by HapX and activation of *HAPX* by Mig1 under low-iron conditions and Mig1 regulation of mitochondrial functions, including respiration, tolerance for reactive oxygen species, and expression of genes for iron consumption and iron acquisition functions. Consistently with these regulatory functions, a *mig1*Δ mutant had impaired growth on inhibitors of mitochondrial respiration and inducers of ROS. Furthermore, deletion of *MIG1* provoked a dysregulation in nutrient sensing via the TOR pathway and impacted the pathway for cell wall remodeling. Importantly, loss of Mig1 increased susceptibility to fluconazole, thus further establishing a link between azole antifungal drugs and mitochondrial function. Mig1 and HapX were also required together for survival in macrophages, but Mig1 alone had a minimal impact on virulence in mice. Overall, these studies provide novel insights into a HapX/Mig1 regulatory network and reinforce an association between mitochondrial dysfunction and drug susceptibility that may provide new targets for the development of antifungal drugs.

**IMPORTANCE** Fungal pathogens of humans are difficult to treat, and there is a pressing need to identify new targets for antifungal drugs and to obtain a detailed understanding of fungal proliferation in vertebrate hosts. In this study, we examined the roles of the regulatory proteins Mig1 and HapX in mitochondrial function and antifungal drug susceptibility in the fungus *Cryptococcus neoformans*. This pathogen is a particular threat to the large population of individuals infected with human immunodeficiency virus (HIV). Our analysis revealed regulatory interactions between Mig1 and HapX, and a role for Mig1 in mitochondrial functions, including respiration, tolerance for reactive oxygen species, and expression of genes for iron consumption and iron acquisition functions. Importantly, loss of Mig1 increased susceptibility to the antifungal drug fluconazole, which is commonly used to treat cryptococcal disease*.* These studies highlight an association between mitochondrial dysfunction and drug susceptibility that may provide new targets for the development of antifungal drugs.

## Abstract (precis)

Fungal pathogens of humans are difficult to treat, and there is a pressing need to identify new targets for antifungal drugs and to obtain a detailed understanding of fungal proliferation in vertebrate hosts. In this study, we examined the roles of the regulatory proteins Mig1 and HapX in mitochondrial function and antifungal drug susceptibility in the fungus *Cryptococcus neoformans*. This pathogen is a particular threat to the large population of individuals infected with human immunodeficiency virus (HIV). Our analysis revealed regulatory interactions between Mig1 and HapX, and a role for Mig1 in mitochondrial functions, including respiration, tolerance for reactive oxygen species, and expression of genes for iron consumption and iron acquisition functions. Importantly, loss of Mig1 increased susceptibility to the antifungal drug fluconazole, which is commonly used to treat cryptococcal disease*.* These studies highlight an association between mitochondrial dysfunction and drug susceptibility that may provide new targets for the development of antifungal drugs.

## INTRODUCTION

Invasive fungal infections emerged in the early 1980s as a major cause of life-threatening illness coincident with aggressive anticancer chemotherapy and immunosuppressive diseases, such as HIV/AIDS (1). *Cryptococcus neoformans* is an opportunistic pathogen that causes a lethal fungal meningoencephalitis in immunocompromised individuals, with a particularly severe impact on patients with HIV/AIDS (2). This burden of disease makes *C. neoformans* one of the deadliest pathogens worldwide when combined with HIV (3). Antifungal drugs, such as amphotericin B, flucytosine, and fluconazole, are available to treat cryptococcosis, but the limited number of drugs, their relatively high toxicity, and the emerging resistance to them emphasize the need for new therapeutic approaches and additional drugs (4). In this context, it has recently been demonstrated that mitochondrial-respiration-deficient mutants of *Saccharomyces cerevisiae* are hypersensitive to the antifungal drug caspofungin, suggesting that mitochondrial respiration plays a role in drug susceptibility (5). In addition, mitochondrial dysfunction is associated with virulence and drug susceptibility in human fungal pathogens, making the mitochondrion a potential new target for antifungal therapy (6).

The CCAAT-binding (Hap) complex composed of the Hap2, -3, -4, and -5 proteins is a key regulator of mitochondrial functions in fungi, and this complex has been well characterized in *S. cerevisiae*. The complex coordinates a shift in nuclear and mitochondrial gene expression upon glucose exhaustion to favor transcription of mitochondrial genes, such as those for the cytochrome subunits and enzymes of the tricarboxylic acid (TCA) cycle (7, 8). More broadly, the Hap complex participates in an interconnected regulatory and signaling pathway with Snf1/Mig1, Rgt2/Snf3, cyclic AMP (cAMP)/protein kinase A (PKA), and Sch9 to sense glucose and trigger a pleiotropic transcriptional response for genes involved in alternative carbon utilization, gluconeogenesis, respiration, and β-oxidation (9, 10). Loss of Mig1 causes the derepression of genes involved in the metabolism of alternative carbon sources as well as genes involved in gluconeogenesis and respiration (11, 12). The control of respiration and glucose repression appear to be linked because repression of the targets of Mig1 involved in the metabolism of alternative sugars occurs upon overexpression of *Hap4* in a *mig1*Δ strain (13). It is also believed that Mig1 is active only when glucose concentrations are elevated; however, recent evidence indicates that Mig1 negatively influences the transcription of genes involved in respiration and iron transport under glucose-limited conditions (14). Recently, Yao et al. (15) also demonstrated a role for Mig1 in cells exhibiting elevated proteasome activity that extends the lifespan of *S. cerevisiae*. Cells with enhanced proteasome capacity leading to lifespan extension also showed increased respiratory activity and responsiveness to oxidative stress due to enhanced turnover of Mig1 and its partial relocation in mitochondria. Yao et al. (15) also proposed that cytoplasmic Mig1 positively impacts cellular respiration.

We previously reported that HapX and Hap3 in *C. neoformans* negatively regulate the expression of genes encoding mitochondrial respiratory and TCA cycle functions under low-iron conditions (16). For example, HapX activates the transcription of the genes for the siderophore transporter Sit1, several heme biosynthesis functions, and the iron regulatory GATA factor Cir1, whereas Hap3 had little influence on the regulation of iron acquisition systems. In general, HapX plays both positive and negative roles in modulating transcriptional responses to iron deprivation and regulates mitochondrial functions (16). In this study, we explored the regulatory connections between a candidate Mig1 ortholog and HapX for genes involved in mitochondrial functions. Our functional analyses revealed a role for Mig1 in mitochondrial processes, such as respiration, energy production, heme biosynthesis, and drug resistance. Remarkably, loss of both Mig1 and HapX impaired the survival of fungal cells in macrophages, but Mig1 was not required for virulence in mice.

## RESULTS

### Identification of a candidate *MIG1* ortholog in *C. neoformans*.

**FIG 1:**
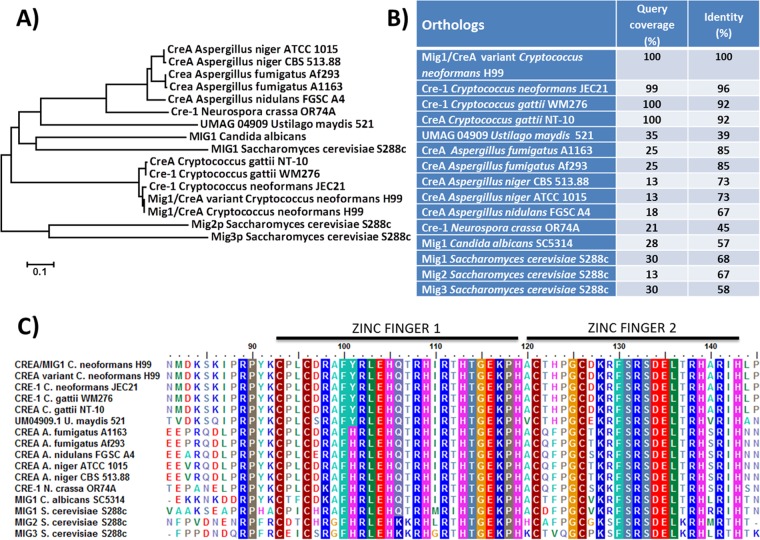
Sequence analysis of *MIG1/CREA/CRE-1*-encoded proteins in fungi. (A) Phylogenic tree of Mig1/CreA/Cre-1 among selected fungi. An unrooted tree was created using the neighbor-joining consensus trees based on the calculated distances using 1,000 bootstrap replications. (B) Amino acid sequence identity between Mig1 of *C. neoformans* H99 and candidate orthologs in selected fungi. (C) Conservation of the zinc finger domains among Mig1 orthologs.

To extend our analysis of regulators of mitochondrial and metabolic functions, we first identified a candidate ortholog of Mig1 by searching the *C. neoformans* genome database with the Mig1 sequence from *S. cerevisiae* (17). A phylogenetic analysis was then performed in our current study to compare the identified sequence (designated *MIG1/CREA* [CNAG_06327]) with the sequences of orthologs from several fungal pathogens of humans, as well as other fungi. This analysis demonstrated conservation of the Mig1 amino acid sequence among strains of *C. neoformans* (JEC21) and *Cryptococcus gattii* (WM276 and NT-10) (Fig. 1A and B). However, the conservation in sequence identity was lower than in other fungal orthologs and occurred mainly in the conserved zinc finger domain consisting of two C_2_H_2_ domains (Fig. 1C). For subsequent analysis of the role of Mig1 in *C. neoformans*, the entire coding region of the gene was deleted with two different selectable markers to generate independent mutants (see Materials and Methods) (17). Initial experiments revealed that the mutants behaved like the wild type (WT) for the formation of the major virulence factors, such as capsule and melanin, as well as in growth at 37°C. Further phenotypic characterization of the deletion mutants and a complemented strain is described below.

### Impact of *MIG1* deletion on the transcriptome.

**FIG 2:**
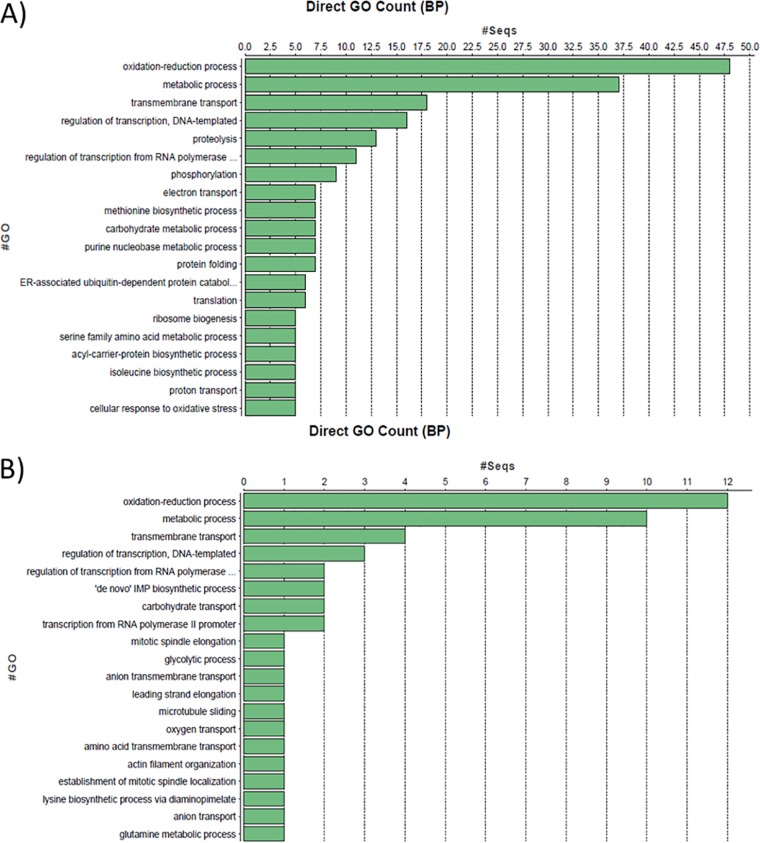
GO term classifications for genes regulated by Mig1. The Blast2Go algorithm was used to classify genes based on the gene ontology (GO) terms for biological processes (BP) found to be significantly regulated by Mig1 in low-iron (A) and iron-replete (B) media.

To begin characterizing the role of Mig1 in *C. neoformans*, we performed a microarray experiment to compare transcript differences for a *mig1*Δ mutant and the WT strain grown in low-iron medium (LIM) with and without ferric chloride. The analysis of differentially expressed genes revealed enrichment for oxidation-reduction and metabolic processes when *mig1*Δ cells were compared to WT cells in media with and without ferric chloride (Fig. 2; see also Table S2 in the supplemental material). Interestingly, a substantial number of genes were found to be differentially expressed in the *mig1*Δ mutant in the absence of iron, thus suggesting a possible iron-related contribution of Mig1. Specifically, we noted that the genes for several oxidoreductase enzymes involved in the metabolism of nucleotides, carbohydrates, amino acids, heme, fatty acids, ergosterol, and folic acid, as well as enzymes involved in cellular respiration, were overrepresented. An enrichment for other genes involved in the transport of organic compounds and ions as well as cell signaling, stress response, cellular division, and gene and protein expression was also observed. We noted that a subset of genes involved in mitochondrial functions was also enriched in the transcriptome of the *mig1*Δ mutant. For example, genes encoding functions for the oxidative decarboxylation of pyruvate, the TCA cycle, the electron transport chain (ETC), and mitochondrial transport were more highly expressed in a *mig1*Δ mutant than in the WT strain when they were grown in LIM (see Table S2 in the supplemental material). Taken together, these results suggested a role for Mig1 in regulating mitochondrial functions and prompted subsequent experiments to investigate the phenotypic impact of deletion of *MIG1* and its relationship with HapX.

### *MIG1* transcript levels are repressed by HapX, and Mig1 positively influences *HAPX* transcript levels in low iron.

**FIG 3:**
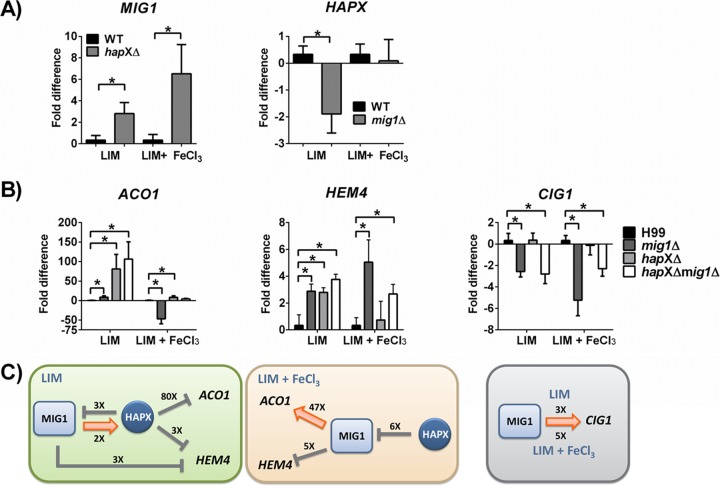
Analysis of *MIG1* and *HAPX* regulatory impact on transcript levels on genes involved in mitochondrial function. (A) Quantitative real-time RT-PCR of *MIG1* and *HAPX* transcript levels in *hapXΔ* and *mig1*Δ mutants compared to WT transcript levels when cells were grown in low-iron and iron-replete (LIM plus 100 µM ferric chloride) media. (B) Transcript levels in *mig1*Δ, *hapX*Δ, and *hapX*Δ *mig1*Δ mutants compared to WT levels for cells grown in LIM and in LIM plus 100 µM FeCl_3_. Transcript levels are shown for *ACO1* (CNAG_01137), encoding a putative mitochondrial aconitase, *HEM4* (CNAG_01908), encoding a uroporphyrinogen III synthase involved in heme biosynthesis, and *CIG1* (CNAG_01653), encoding a putative hemophore involved heme uptake. Experiments were carried out in triplicate. Values are reported as the means ± standard errors of the means (SEM). Statistical significance was calculated using the unpaired two-tailed *t* test (*, *P* < 0.05). (C) Schematic representations of proposed regulatory interactions between HapX and Mig1 and the influence of these regulators on transcript abundances of *ACO1*, *HEM4*, and *CIG1*. The numbers indicate the fold changes in transcript levels.

We previously demonstrated a role for HapX in the repression of iron-dependent genes encoding components of the electron transport chain and the TCA cycle under low-iron conditions (16). Given that deletion of *MIG1* altered the expression of genes for related processes, we next tested whether HapX influenced the expression of *MIG1* under low-iron and iron-replete conditions. The relative levels of expression of *MIG1* were compared between the WT strain and a *hapX*Δ mutant by quantitative reverse transcription-PCR (qRT-PCR), and we found that deletion of *HAPX* increased the transcript level of *MIG1* in every growth condition regardless of the iron state (Fig. 3A). On the other hand, *HAPX* transcript levels were significantly reduced in a *mig1*Δ mutant compared to those in the WT when cells were grown in LIM, and no difference in expression was measured when cells were grown in iron-replete medium (Fig. 3A). This outcome suggested that Mig1 positively influences *HAPX* transcription under the low-iron condition and thus may contribute to the activity of HapX to limit the expression of iron-requiring functions in this situation. Taken together, these observations revealed reciprocal regulation of *MIG1* and *HAPX* transcript levels, particularly under the low-iron condition.

### Shared and distinct regulatory influences of Mig1 and HapX.

The regulatory influence of Mig1 and its relationship with HapX prompted a further investigation of the impact of loss of Mig1 on the expression of a set of HapX-regulated genes. These genes encoded mitochondrion-associated functions, such as aconitases, as well as enzymes involved in amino acid and heme biosynthesis and the TCA cycle. We used quantitative RT-PCR to first test the impact of *MIG1* deletion on the transcript level of the aconitase gene *ACO1* (Fig. 3B). We found that Mig1 exerts a minor negative influence on *ACO1* transcript abundance under the low-iron condition and a large positive influence in the presence of iron (Fig. 3B). We had previously demonstrated that HapX negatively influences the transcript level of *ACO1* and another aconitase gene (*ACO2*) during iron limitation (16). As shown in Fig. 3B, we confirmed this regulatory influence of HapX on *ACO1* and found that a *hapX*Δ *mig1*Δ double mutant behaved like the *hapX*Δ mutant. Our microarray analysis with the *mig1*Δ mutant revealed that another aconitase gene, *ACO4*, and the *LEU1* gene were also regulated by Mig1 (see Table S2 in the supplemental material). Leu1 and the aconitases require Fe−S clusters for their enzymatic activities, and as such, they are targets of repression by HapX when iron is limited. Our group also recently showed that transcript abundance for *LEU1* is negatively influenced by HapX under the low-iron condition (18). To add depth to our analysis, we confirmed Mig1 regulation of *LEU1*, *ACO4*, and two other aconitase genes (*ACO2* and *ACO3*) by qRT-PCR (Fig. S2). To summarize the observed regulation, Fig. 3C presents a possible regulatory scheme for the direct or indirect influences of Mig1 and HapX on each other and on the *ACO1* gene. HapX in particular has a negative influence on *ACO1* under the low-iron condition, and Mig1 positively regulates *ACO1* when iron is available.

Several genes involved in heme biosynthesis in *C. neoformans* were previously found to be regulated by HapX, including *HEM4*, which encodes uroporphyrinogen III synthase (16). We therefore tested whether Mig1 also exerts a regulatory influence on *HEM4* and found that transcripts for the gene were more abundant under iron-depleted and -replete conditions in the *mig1*Δ mutant than in the WT (Fig. 3B). This pattern of regulation is similar to the one observed in the transcriptome analysis of the *hapX*Δ mutant (16), and indeed, the qRT-PCR analysis with the *hapX*Δ mutant revealed elevated *HEM4* transcript levels under the low-iron condition (Fig. 3B). Under this condition, the *hapX*Δ *mig1*Δ double mutant also showed elevated *HEM4* transcript abundance, as seen with either of the single mutants (Fig. 3B). In contrast, loss of HapX had little influence on *HEM4* transcript levels under the iron-replete condition, and the double mutant behaved similarly to the *mig1*Δ single mutant. Figure 3C illustrates possible regulatory schemes in which Mig1 exerts a negative influence on *HEM4* transcript levels under both iron conditions, and HapX participates in negative regulation only under the low-iron situation. The influence of Mig1 on *HEM4* upon iron limitation may occur via an influence on HapX expression or independently, as shown in Fig. 3C. It should be noted that we also used qRT-PCR to confirm Mig1 regulation of a second heme biosynthetic gene, *HEM3*, as predicted by our microarray analysis (Fig. S2; Table S2).

The regulation of heme biosynthesis genes by Mig1 is interesting because we have previously shown that *C. neoformans* uses heme as an iron source (16, 19, 20). We therefore tested whether Mig1 influenced transcript levels for the highly iron-responsive gene *CIG1*, which encodes a putative heme-binding protein (19). Our analysis demonstrated a significant decrease in the transcript levels for the *CIG1* gene in the *mig1*Δ mutant compared to those in WT cells grown in low-iron or iron-replete medium (Fig. 3B). A similar influence of Mig1 was seen for the *SIT6* gene, encoding a putative siderophore transporter (see Fig. S2 in the supplemental material). We did not observe a regulatory influence of HapX on *CIG1* transcript abundance regardless of the iron level (Fig. 3B). Additionally, the regulatory pattern for the *hapX*Δ *mig1*Δ double mutant was similar to that of the *mig1*Δ mutant, further indicating that Mig1 has a distinct regulatory influence, as diagrammed in Fig. 3C. Taken together, the quantitative RT-PCR results suggest that Mig1 participates in a regulatory network with HapX to influence the expression of iron-utilizing (i.e., the aconitases and Leu1) and heme biosynthetic functions in response to iron availability. Additionally, Mig1 exerts a regulatory influence on some iron-responsive genes, like *CIG1*, over which HapX has little influence.

### Mig1 is required for resistance to inhibitors of the electron transport chain and ROS tolerance.

**FIG 4:**
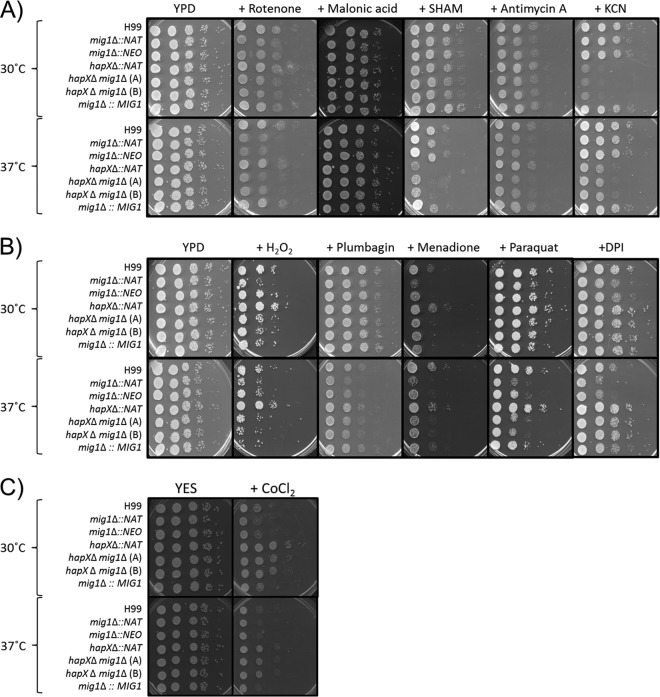
The *mig1*Δ and *hapX*Δ mutants have altered susceptibilities to inhibitors of cellular respiration and reactive oxygen species. The results of spot assays are shown for serial dilutions of the WT and derivative mutants under the following conditions. (A) YPD plus inhibitors of electron transport chain complexes I to IV and the alternative oxidase (75 µg ⋅ ml^−1^ rotenone, 1 mM malonic acid, 3 µg ⋅ ml^−1^ antimycin A, 10 mM potassium cyanide [KCN], and 5 mM SHAM). (B) YPD plates plus ROS inducers (0.01% H_2_O_2_, 50 µM plumbagin, 5 µg ⋅ ml^−1^ menadione, 500 µM paraquat, and 50 µM DPI). Plates were incubated at 30°C and 37°C for 2 days. (C) YES plus hypoxia-mimicking agent cobalt-chloride (600 µM CoCl_2_). Plates were incubated at 30°C or 37°C for 2 days.

Our analysis of the influence of *MIG1* deletion on the transcriptome revealed enrichment for genes involved in cellular respiration. This process occurs in mitochondrial cristae and includes the four complexes (I to IV) of the electron transport chain (ETC) that shuttle electrons from NADH and succinate to generate a proton gradient to ultimately produce ATP and reduce oxygen to water (21, 22). The alternative oxidase (Aox1) of the alternate electron transport pathway has also been characterized in *C. neoformans* (23). Given the observed regulation by Mig1, we hypothesized that specific inhibitors of electron transport would impair the growth of the *mig1*Δ mutants. For example, complex I (NADH dehydrogenase), complex III (cytochrome *c* reductase), and complex IV (cytochrome *c* oxidase), which require iron and iron-sulfur clusters to successfully transfer electrons, are inhibited by rotenone, antimycin A, and potassium cyanide (KCN), respectively. Additionally, complex II (succinate dehydrogenase) and the alternative oxidase (Aox1) are impaired by malonic acid and salicylhydroxamic acid (SHAM), respectively (23 – 25). Spot assays revealed that rotenone and antimycin A reduced the growth of the *mig1*Δ mutants, while potassium cyanide and SHAM affected the growth of mutants lacking *hapX*Δ in comparison to the growth of the WT strain. The growth of the *hapX*Δ *mig1*Δ double mutants was impaired by each of the inhibitors, and growth inhibition was generally more prominent at 37°C (Fig. 4A). We noted that exposure to malonic acid, an inhibitor of complex II, did not impair the growth of any mutant. Together, these results indicate that Mig1 influences the electron transport chain at the levels of complexes I and III but that HapX acts on complex IV and the alternative oxidase. Additional work is needed to investigate why an influence on complex II was not detected.

The activity of the mitochondrial respiratory chain produces superoxide (O_2_^•−^) as a primary reactive oxygen species (ROS) at complexes I and III (24). Given the susceptibility of the *mig1*Δ mutants to the inhibitors of these complexes (rotenone and antimycin A, respectively), we predicted that exposure to ROS (H_2_O_2_) or agents that stimulate ROS production (e.g., plumbagin, menadione, and paraquat) would impair the growth of *mig1*Δ mutants. This was indeed the case for the growth of the mutants at 30°C and, as with the inhibitors of electron transport, the growth defect was exacerbated at 37°C (Fig. 4B). In general, these results suggest that Mig1 is required for ROS resistance and/or detoxification. These phenotypes were generally not observed for the *hapX*Δ mutant, and we conclude that Mig1 appears to have a distinct role in mitochondrial cellular respiration at the level of complexes I and III.

To investigate the connection between Mig1 and complex I in more detail, we also tested the susceptibility of the *mig1*Δ mutants to diphenyleneiodonium (DPI), an inhibitor of mitochondrial NADH dehydrogenase in complex I, and other flavoproteins. As shown in Fig. 4B, the *mig1*Δ mutants showed growth inhibition on DPI consistent with the observations with the complex I inhibitor rotenone (Fig. 4A). However, the growth of the *hapX*Δ *mig1*Δ mutants was not impaired on DPI, in contrast to the situation with rotenone, suggesting that additional factors may be involved in the susceptibility of the *mig1*Δ mutants. These factors may involve regulatory interactions between Mig1 and HapX and/or the ability of DPI to influence ROS production.

Mitochondria consume oxygen to fulfill metabolic functions for energy production, as it is the final electron acceptor at complex IV of the ETC. Therefore, growth with low-oxygen tension (hypoxia) affects cellular respiration and dysregulates the expression of genes encoding mitochondrial oxygen-dependent functions (such as the cytochrome subunits) and oxidases and desaturases required for heme, sterol, and fatty acid biosynthesis (7, 26). Interestingly, the *mig1*Δ mutants were sensitive to the hypoxia-mimetic compound cobalt-chloride (CoCl_2_), but deletion of *HAPX* in these mutants abolished this growth inhibition (Fig. 4C). As with the results for DPI, the experimental outcome with CoCl_2_ is consistent with regulatory interactions between Mig1 and HapX (discussed further below).

### Loss of Mig1 increases susceptibility to fluconazole.

**FIG 5:**
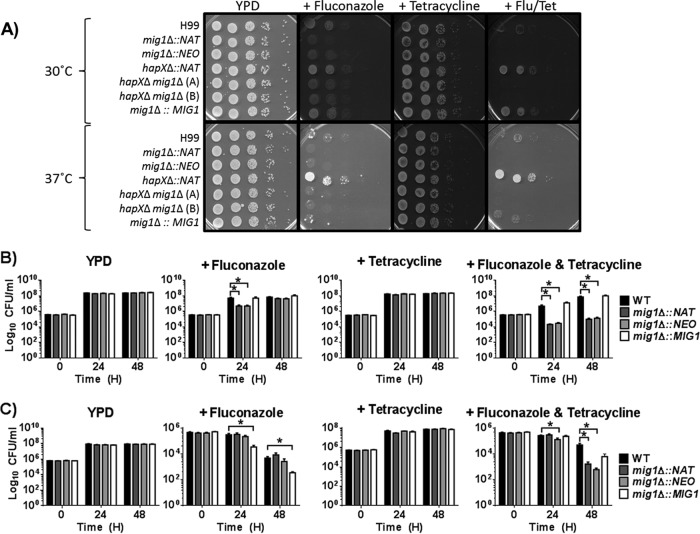
The *mig1*Δ mutant has enhanced susceptibility to fluconazole. (A) Spot assay of serially diluted WT cells of strain H99 and derivative mutants on YPD plates with 10 µg ⋅ ml^−1^ fluconazole (Flu) and/or 100 µg ⋅ ml^−1^ tetracycline (Tet). The plates were incubated at 30°C for 2 days. (B and C) Survival of WT strain H99 and derivative *mig1*Δ mutants grown in YPD liquid media with and without fluconazole and/or tetracycline at 30°C (B) or 37°C (C) with agitation. The numbers of cells per milliliter of each culture were counted at 0, 24, and 48 h, and the means ± SEM were calculated. The experiment was performed twice with biological triplicates. Statistical analysis was performed using an unpaired two-tailed *t* test, where *P* is <0.05 (*).

Iron deficiency and reduced mitochondrial respiration are known to be associated with increased sensitivity to fluconazole in *C. neoformans* (27, 28). Given the influence of the *mig1*Δ mutation on mitochondrial function and the transcript levels of genes for iron acquisition, we hypothesized that the mutant would show altered susceptibility to fluconazole. Indeed, the growth of the *mig1*Δ mutants but not the *hapX*Δ mutant was impaired on medium with fluconazole (Fig. 5A). Furthermore, a complete inhibition of the growth of the *mig1*Δ mutants was achieved with the addition of tetracycline to the medium with fluconazole, whereas tetracycline exposure alone had no effect on the *mig1*Δ mutants. Tetracycline forms complexes with magnesium and inhibits mitochondrial protein synthesis, presumably due to the structural similarity between bacterial and mitochondrial ribosomal machineries (29, 30). Resistance was observed for the *hapX*Δ mutant upon exposure to fluconazole and tetracycline, suggesting further distinct roles for Mig1 and HapX. To examine the connections between Mig1 and mitochondrial function in more detail, we tested the survival of the mutants in liquid medium in the presence of fluconazole, tetracycline, and both drugs. As shown in Fig. 5B, the survival of the *mig1*Δ mutants was severely compromised after 24 h of exposure to both drugs, given that more that 92% of *mig1*Δ mutant cells in the initial inoculum were killed. Further incubation at 30°C resulted in an increase in cell numbers back to approximately 40% of the initial concentration, perhaps due to adaptation or selection of heteroresistant cells. The pattern was different at 37°C, at which temperature fungicidal activity was observed for both fluconazole and the fluconazole-plus-tetracycline combination at 48 h in all strains. Accentuated killing of the *mig1*Δ mutants was observed only with fluconazole and tetracycline compared to the killing of the WT strain at 48 h. Overall, it appeared that the combination of tetracycline and fluconazole was fungicidal for the *mig1*Δ mutants at both 30°C and 37°C.

### Connections between Mig1 and the Tor pathway.

**FIG 6:**
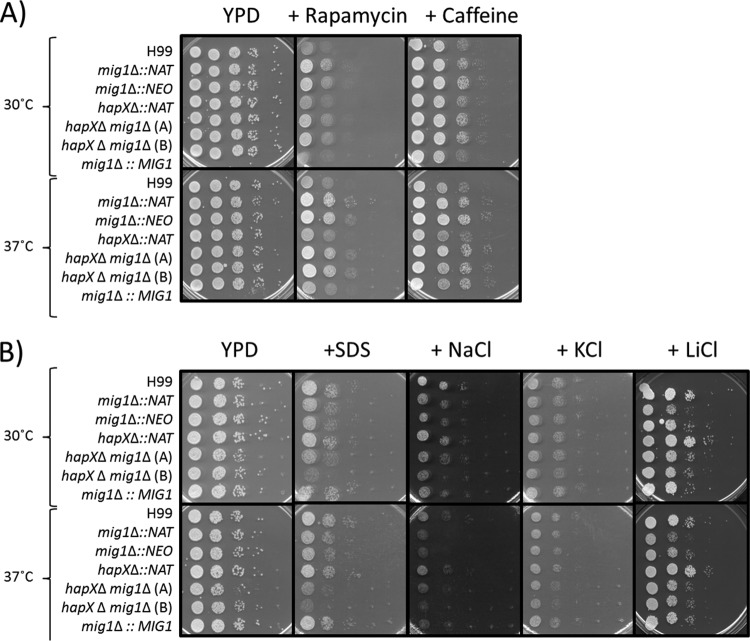
Mig1 influences the TOR and cell wall remodeling pathways. (A) Resistance of the *mig1*Δ mutant to rapamycin/sirolimus and caffeine. Spot assay of serially diluted WT strain H99 and derivative mutants on YPD plates with 10 µg ⋅ ml^−1^ sirolimus and 0.5 mg ⋅ ml^−1^ caffeine. (B) Growth inhibition of the *mig1*Δ mutants by agents that challenge cell wall integrity and by elevated concentrations of salts. Spot assay of serially diluted WT strain H99 and derivative mutants on YPD plates with 0.125% SDS, 1.5 M sodium chloride (NaCl), 1.5 M potassium chloride (KCl), or 100 mM lithium chloride (LiCl). The plates were incubated at 30°C or 37°C for 2 days.

In *C. neoformans*, the TOR and protein kinase C (PKC) signaling pathways are involved in fluconazole tolerance (31). The TOR pathway in *S. cerevisiae*, which involves the complexes TORC1 and TORC2, controls cell growth and the response to nutrient limitation (i.e., nitrogen and carbon sources) (32, 33). The TOR pathway is the target of the drug sirolimus (rapamycin), which acts by binding to the cytosolic immunophilin Fpr1 and interacting with TORC1 to cause inhibition (34 – 37). The kinase Tor1, a component of TORC1, is a target of caffeine, whose inhibition activates the Pkc1p-Mpk1p cascade; caffeine-dependent phenotypes are caused mainly by inhibition of Tor1p. Because deletion of *MIG1* impacted mitochondrial respiration and fluconazole susceptibility, we tested whether there was a connection with the Tor pathway*.* Growth assays revealed enhanced resistance to sirolimus and caffeine upon deletion of *MIG1*, compared to the resistance of the WT strain or the *hapX*Δ mutant, suggesting an impact on Tor1, the TORC1 complex, or downstream targets in a *mig1*Δ mutant (Fig. 6A). In general, these results demonstrate a link between mitochondrial dysfunction caused by the deletion of *MIG1* and the function of the Tor pathway in *C. neoformans.*

The PKC signaling pathway is responsible for cell wall remodeling and the response to various stresses, such as high cation concentrations, that challenge cell wall integrity (38). Because this pathway is also involved in fluconazole susceptibility, we tested whether deletion of *MIG1* affects cell wall integrity. In this context, we found growth defects for the *mig1*Δ and *hapX*Δ *mig1*Δ mutants on sodium dodecyl sulfate (SDS), which indicates compromised cell wall integrity (Fig. 6B). Similarly, the *mig1*Δ and *hapX*Δ *mig1*Δ mutants had growth defects on media containing high concentrations of sodium chloride, potassium chloride, and lithium chloride (Fig. 6B). These phenotypes were exacerbated at 37°C compared to those at 30°C. Overall, these results suggest that Mig1 is required to maintain cell wall integrity, and this connection may partially explain the increased susceptibility to fluconazole upon deletion of *MIG1*.

### Mig1 and HapX are required together for survival in macrophages.

**FIG 7:**
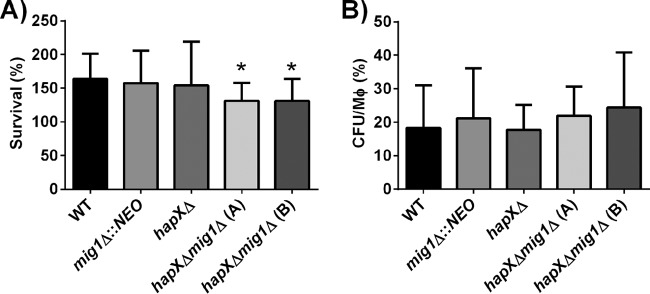
Impaired survival of the *hapX*Δ *mig1*Δ mutants upon phagocytosis. (A) Survival of the WT strain H99 and mutants in the murine-macrophage-like cell line J774A.1 at 24 h p.i. (B) Number of intracellular cryptococcus cells per macrophage (Mφ) after 2 h of interaction. The experiment was performed five times with three biological replicates for each experiment, and the values are expressed as means ± SEM. Values for the double *hapX*Δ *mig1*Δ(A) and *hapX*Δ *mig1*Δ(B) mutants were statistically significantly different from values for the WT using a two-tailed unpaired *t* test (*, *P* ≤ 0.05).

Alveolar macrophages are the first line of innate defense when cryptococcal cells enter vertebrate hosts. A microarray study of infected murine-macrophage-like J774A.1 cells with *C. neoformans* strain H99 demonstrated activation of genes encoding multiple membrane transporters for hexoses, amino acids, and iron, as well as genes involved in responses to oxidative stress, autophagy, peroxisome function, Ca^2+^/calcineurin signaling, and lipid metabolism (39). Our expression analysis revealed that several of these genes and processes were also affected by deletion of *MIG1*. Therefore, we assessed the survival of the *mig1*Δ, *hapX*Δ, and *hapX*Δ *mig1*Δ mutants in the murine-macrophage-like cell line J774A.1. Intracellular survival and proliferation of the single *mig1*Δ and *hapX*Δ mutants were similar to those of the WT, but impaired intracellular survival was found for the *hapX*Δ *mig1*Δ double mutants at 24 h postinfection (Fig. 7A). The strains all showed similar levels of uptake at 2 h (Fig. 7B). Quantitative RT-PCR of the *HAPX* and *MIG1* transcript levels after 24 h of intracellular proliferation in macrophages compared to levels in cells grown in Dulbecco’s modified Eagle’s medium (DMEM) under similar conditions revealed a downregulation of *MIG1* (data not shown). Taken together, these analyses suggest that a balance of Mig1 and HapX may support intracellular survival upon phagocytosis.

### Mig1 is not required for virulence in a murine inhalation model of cryptococcosis.

**FIG 8:**
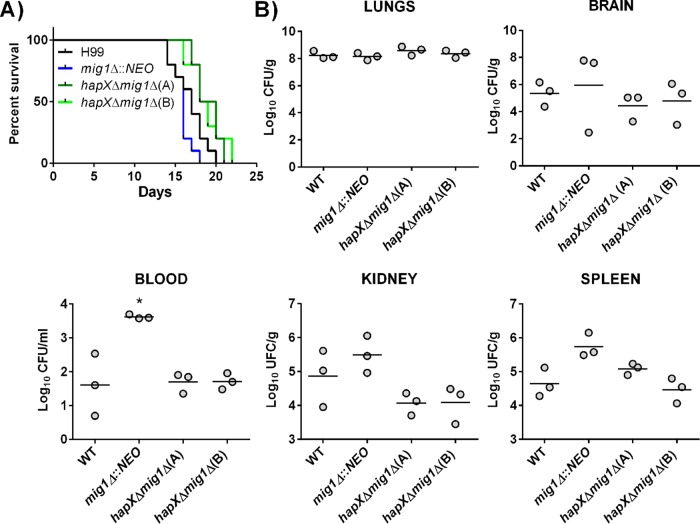
The *hapX*Δ *mig1*Δ mutant but not the *mig1*Δ mutant is attenuated for virulence in mice. Ten female BALB/c mice were infected intranasally with either the WT strain, the *mig1*Δ mutant, or the *hapX*Δ *mig1*Δ mutants, and survival of the mice was monitored over 22 days. (A) The difference in survival between the *mig1*Δ mutant-infected and the WT-infected mice was not significant, but a significant difference was statistically reached between values for mice infected with the WT and the *hapX*Δ *mig1*Δ(A) mutant (*P* < 0.05) and between values for mice infected with the *mig1*Δ, *hapX*Δ *mig1*Δ(A), and *hapX*Δ *mig1*Δ(B) mutants (*P* < 0.0001) based on the log-rank Mantel-Cox test. (B) Fungal burden was determined in systemic organs and cardiac blood for three mice infected with the WT strain, the *mig1*Δ mutant, the *hapX*Δ *mig1*Δ mutants, or the *mig1*Δ::*MIG1* mutant at the time of death (*, *P* < 0.05). The Mann-Whitney *U* test was used for statistical analysis.

**FIG 9:**
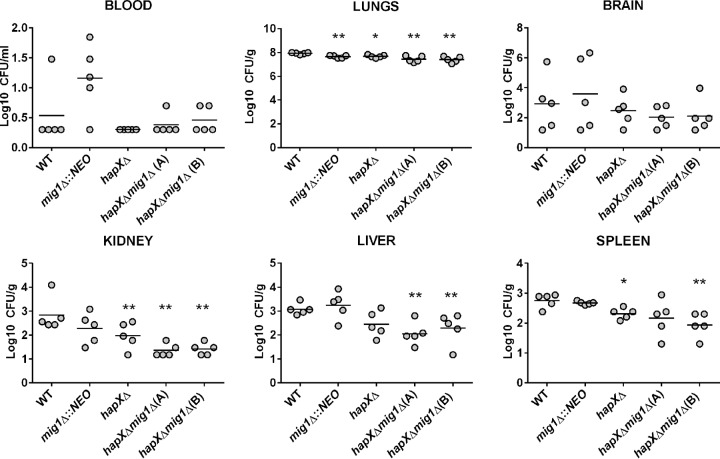
The *hapX*Δ *mig1*Δ mutant is significantly attenuated for virulence in mice after 15 days of infection. Ten female BALB/c mice were infected with the WT strain, the *mig1*Δ mutant, or the *hapX*Δ *mig1*Δ mutants and sacrificed at 15 days postinfection. Cardiac blood and systemic organs were aseptically removed to assess fungal burden. The differences in fungal loads between mice infected with the *mig1*Δ mutant and the WT were not significant in any organs (except the lungs), but a statistically significant difference was reached between values for mice infected with the WT and the *hapX*Δ *mig1*Δ(A) and *hapX*Δ *mig1*Δ(B) mutants in systemic organs (lungs, kidney, liver, and spleen). Statistical significance was achieved using the two-tailed unpaired Mann-Whitney test. *, *P* < 0.05; **, *P* < 0.01.

We next assessed the virulence of the mutants in a murine inhalation model of cryptococcosis and found that a *mig1*Δ mutant is able to establish an infection and cause disease similar to that caused by the WT strain. Interestingly, mice infected with the *mig1*Δ mutant developed disease-related symptoms somewhat earlier than those infected with the WT strain, although the difference was not statistically significant (Fig. 8A). However, deletion of *HAPX* in a *mig1*Δ mutant delayed symptom formation by 2 to 3 days, but this difference is likely attributable solely to *HAPX* because a *hapX*Δ mutant showed similar attenuation in a previous study (16). An examination of the fungal burden for each strain at the time of sacrifice revealed comparable levels of mutant and WT cells in each tested organ or tissue, with the exception of blood (Fig. 8B). In blood, the loss of Mig1 appeared to improve survival or proliferation. The impact of deletion of *MIG1*, *HAPX*, or both genes on fungal burden was also examined at a fixed time point of 15 days postinfection for all of the animals. We found that the burden of the *mig1*Δ mutant was slightly lower in the lungs than the burden of the WT, and the *hapX*Δ mutation had some influence on burdens in the lung, kidney, and spleen. However, the burdens of the *hapX*Δ *mig1*Δ double mutants were significantly reduced in the lungs, liver, kidney, and spleen (one of the two mutants) but, interestingly, not in the brain (Fig. 9). We again noted a trend toward higher accumulations of the *mig1*Δ mutant in blood, but there was considerable variability between mice in this experiment. Overall, this analysis indicated that both *HAPX* and *MIG1* participate in the elaboration of the virulence composite of *C. neoformans*, but they had no significant individual impact on disease.

## DISCUSSION

There is an urgent need for new antifungal agents because of emerging resistance and issues of efficacy and toxicity for the current antifungal arsenal (4, 40). It has been proposed that the mitochondrion represents an excellent target for antifungal drug development because its dysfunction is associated with virulence and drug susceptibility in fungal pathogens (6). In this context, our ongoing analysis of the regulatory network for iron homeostasis in *C. neoformans* revealed a role for the candidate transcription factor Mig1 in mitochondrial function and fluconazole susceptibility. Specifically, our transcriptome analysis indicated that loss of Mig1 influenced transcript levels for mitochondrial functions, including the biosynthesis of amino acids, carboxylic acids, and heme, as well as TCA cycle and electron transport chain components. Additionally, *mig1*Δ mutants show enhanced susceptibility to inhibitors of ETC complexes I and III. The growth of *mig1*Δ mutants was also influenced by perturbation of ROS levels, thus indicating a role for Mig1 in the response to oxidative stress in the context of mitochondrion electron transport as a major source of ROS. This impact of Mig1 on mitochondrial functions is interesting in light of our finding that the *mig1*Δ mutants also have increased susceptibility to fluconazole, and this study strengthens the connection between azoles and mitochondrial functions in *C. neoformans*.

We focused on the role of Mig1 and identified both shared and distinct contributions of this candidate transcription factor with the HapX regulatory subunit of the Hap complex. We previously characterized HapX as a transcriptional regulator of mitochondrial function in the context of iron homeostasis (16). Our current comparison was motivated by our transcriptome studies that established connections between the Hap complex, mitochondria, iron homeostasis, and susceptibility to antifungal drugs in *C. neoformans* (16, 27, 28). We have shown before that HapX has a conserved regulatory function distinct from that of the Hap4 protein of *S. cerevisiae* but similar to that of *Aspergillus nidulans* HapX with regard to repression of iron-dependent functions during iron limitation (16, 41). Because *MIG1* promoted transcription of *HAPX* under low-iron conditions, we tested whether Mig1 had an impact on the transcription of iron-responsive genes previously identified as regulated by HapX. We indeed found that Mig1 regulates the transcript levels of a subset of genes encoding iron-consuming functions, such as aconitases and heme biosynthetic enzymes, and also regulated a gene for iron acquisition (Fig. 3). Interestingly, elevated transcripts were also observed for *MIG1* in a *hapX*Δ mutant when cells were grown under either low-iron or iron-replete conditions. HapX may therefore directly or indirectly repress *MIG1* transcription, perhaps through the activity of the Hap2/3/5 complex. Overall, these results suggest intriguing connections between these two transcription factors to coordinate the expression of genes for mitochondrial functions and iron availability. Possible scenarios for connections between HapX and Mig1 in the regulation of target genes are proposed in Fig. 3C.

The regulatory influences of Mig1 and HapX on each other explain, at least in part, why the *mig1Δ* and *hapXΔ* mutants had opposite phenotypes with regard to susceptibilities to ETC inhibitors, ROS, fluconazole-tetracycline, sirolimus, caffeine, and agents that challenge cell wall integrity. In the majority of cases, the double *hapX*Δ *mig1*Δ mutant had the phenotype of the *mig1Δ* mutant. This observation is consistent with the observed repressive effect of HapX on *MIG1* transcript levels and the participation of these factors in a shared regulatory network. However, it is also possible that some of the phenotypes arose from separate regulatory influences of Mig1 and HapX or a negative influence of Mig1 on HapX.

Two notable exceptions in which the *hapX*Δ *mig1*Δ double mutant had the phenotype of the *hapX*Δ mutant were found, and these involved exposure to DPI, an inhibitor of mitochondrial NADH-ubiquinone oxidoreductase and other flavoproteins, and the hypoxia-mimicking agent cobalt-chloride (CoCl_2_). The *mig1*Δ mutants were sensitive, and the *hapX*Δ and double mutants were resistant. One interpretation is that under the rich-medium conditions employed for the assays with DPI and CoCl_2_ (i.e., yeast extract-peptone-dextrose [YPD] and yeast extract-sucrose [YES], respectively), Mig1 negatively regulates HapX to allow a wild-type level of growth. Loss of Mig1 in the *mig1*Δ mutant would allow an increased influence of HapX, leading to sensitivity, and deletion of HapX in the *hapX*Δ mutant would reduce its influence and confer resistance. The absence of HapX in the double mutant explains the observed resistance (Fig. 4B and C). With regard to a Mig1-HapX regulatory network, we previously found that reduced expression of genes for respiration functions correlated with increased fluconazole susceptibility (as also seen for the *mig1Δ* mutant), and this association was further strengthened by the finding that DPI increased azole susceptibility (27). We also found that the *hap3* mutant but not the *hapX* mutant had decreased susceptibility to azole drugs, and we predicted that additional regulatory factors were involved. Mig1 appears to be one of these additional regulatory factors because loss of Mig1 but not HapX accounted for the drug susceptibility.

The antifungal activity of fluconazole is due to inhibition of two heme-containing cytochrome P450s involved in lanosterol 14α-demethylation (Erg11) and Δ22 desaturation (Erg3) (42). Inhibitions result in a block in ergosterol biosynthesis with accumulation of toxic sterol intermediates and the disruption of membrane integrity (43). Given that ergosterol synthesis requires oxygen and that the active sites of Erg11 and Erg3 contain heme, iron homeostasis and mitochondrial functions, such as heme biosynthesis and respiration, have an impact on ergosterol biosynthesis. In fact, mutants with mutations in proteins for mitochondrial functions of *Candida albicans* have altered cellular sterol compositions (44, 45). Furthermore, a connection between iron deficiency and increased sensitivity to fluconazole has previously been reported for *C. neoformans* (20, 27). The impact of iron deficiency is clear because we have shown that deletion of the *CFO1* gene, encoding the ferroxidase for high-affinity iron uptake, results in downregulation of a set of ~30 genes encoding mitochondrial functions (28). Given that deletion of *MIG1* dysregulates cellular respiration and impacts genes for iron-consuming functions in an iron-dependent manner, it was not surprising to see an increased sensitivity to fluconazole. We speculate that this sensitivity can in part be explained by an altered sterol production and membrane function because other studies of *C. neoformans* have shown that azole tolerance involves the sterol response element-binding protein Sre1, as its deletion confers azole sensitivity (46, 47). Furthermore, no growth defect was observed when *mig1*Δ mutants were exposed to the polyene drug amphotericin B (data not shown), which supports a specific role of Mig1 in fluconazole susceptibility.

In fact, the fluconazole susceptibility for the *mig1*Δ mutants was fungicidal when combined with the mitochondrial translation inhibitor tetracycline at physiological temperature. This result is consistent with that of a previous study of the DEAD box RNA helicase Vad1 and the mitochondrial elongation factor Tuf1 that revealed a role for mitochondrial protein synthesis in the regulation of fluconazole susceptibility in *C. neoformans*. For example, a *vad1* deletion mutant has impaired growth on medium containing respiration-dependent carbon sources, an increased resistance to fluconazole, and inhibition of translation of *TUF1*, suggesting a defect in mitochondrial function. On the other hand, overexpression of *TUF1* in a *vad1* mutant rescued growth on glycerol, pyruvate, and lactate and resulted in an increase in fluconazole sensitivity in combination with tetracycline compared to that of the WT. Overall, these studies strongly suggest a role for mitochondrial respiration in fluconazole susceptibility that is reinforced by prior observations (48, 49). Several mechanisms of tetracycline resistance/detoxification, including efflux pumps, modification of ribosomal protection, rRNA mutation, and degradation of tetracycline, exist in bacteria. This last mechanism is particularly interesting because it involves FAD-requiring monooxygenases, which utilize NADPH and O_2_ to modify tetracycline and alter its ability to bind magnesium and thus presumably reduce the drug affinity to its target, the ribosome. The modified tetracycline subsequently undergoes nonenzymatic degradation. The requirement of O_2_ for the monooxygenase activity means that the resistance mechanism is coupled to cellular respiration (30). For our results, perturbation of cellular respiration caused by the deletion of *MIG1* in *C. neoformans* increased fluconazole susceptibility in combination with tetracycline, presumably by dysregulating iron homeostasis and oxygen consumption by the ETC and thus impacting ergosterol biosynthesis.

The TOR and PKC signaling pathways are required for basal tolerance to fluconazole in *C. neoformans* (31). Because deletion of *MIG1* increased resistance to sirolimus and caffeine, which inhibit components of the TOR pathway, it was not surprising to observe fluconazole susceptibility. If we use *S. cerevisiae* as a model, it is known that cross talk occurs between the TOR pathway (composed of TORC1 and TORC2) and glucose sensing by the AMPK/Snf1 pathway, which in turn regulates Mig1 in yeast. TORC1 is active under optimal growth conditions and promotes ribosome biogenesis, protein translation, and cell growth, while its activity is inhibited upon nutritional limitation and stress (50). The AMPK/Snf1 pathway is then activated under these conditions (10). Given the opposite roles of these nutrient-sensing pathways, we can speculate that deletion of *MIG1* in *C. neoformans* may activate the Tor pathway perhaps by interfering with the negative influence of the glucose-sensing pathway. Interestingly, deletion of *TOR1* in *S. cerevisiae* results in an increase in mitochondrial respiration by enhancing the translation of mitochondrial-DNA-encoded oxidative phosphorylation proteins. Therefore, it has been proposed that inhibition of TOR signaling causes derepression of respiration during growth in glucose (51, 52). Recently, a comparative proteomic study of *Aspergillus fumigatus* identified the protein kinase Tor to be involved in mitochondrial functions, such as respiration and the biosynthesis of ornithine/arginine, a precursor of siderophore formation. The abundances of proteins involved in mitochondrial respiration vary from those in the WT upon Tor kinase repression. The authors also demonstrated that induction of ornithine biosynthesis under an iron starvation condition was lost when *tor* expression was repressed, and this was independent of HapX (53). Future work on these intricate pathways will extend our comprehension of the role of Mig1 and HapX beyond iron homeostasis and cellular respiration to include a study of nutrient sensing and the AMPK/Snf1 pathway. For example, glucose exerts a negative regulation on the Hap complex in some fungi, and in turn, the complex regulates several mitochondrial processes involving iron consumption pathways (54, 55).

Our analysis of virulence for the *mig1*Δ mutant revealed levels of intracellular survival in macrophages similar to those of the WT strain. We speculate that this intracellular survival can be attributed to the fact that the *mig1*Δ mutant may be insensitive to external signals and already primed to survive in the harsh intracellular environment of a macrophage. In this context, a microarray study of the *C. neoformans* strain H99 transcriptome upon infection of murine-macrophage-like J774A.1 cells demonstrated activation of genes encoding multiple membrane transporters for hexoses, amino acids, and iron, as well as genes for oxidative stress, autophagy, peroxisome function, Ca^2+^/calcineurin signaling, and lipid metabolism (39). We found that deletion of *MIG1* influenced similar processes. However, deletion of *HAPX* in a *mig1*Δ mutant (but not loss *mig1*Δ alone) impaired intracellular survival, suggesting that the combined regulatory contributions of HapX and Mig1 support intraphagolysosome survival. Similarly, in mice, a *mig1*Δ mutant was able to establish an infection and cause disease at the same level as the WT, although mice infected with the *mig1*Δ mutant developed symptoms slightly earlier than the WT. The difference was not statistically significant, although the slight hypervirulence of the *mig1*Δ mutant was observed in a second independent experiment (data not shown). However, only the deletion of *HAPX* in a *mig1*Δ mutant significantly reduced virulence, but this difference was likely attributable solely to HapX, as previously determined for this single mutant (16). Our evaluation of the fungal burden at 15 days postinfection confirmed that yeast cells of the double *hapX*Δ *mig1*Δ mutants were significantly reduced in systemic organs (lungs, liver, spleen, and kidney), indicating impaired virulence for this double mutant compared to that of the WT strain. Taken together, this illustrates that Mig1 alone has negligible impact on the virulence composite and that instead there is a requirement for a combined regulatory influence from both Mig1 and HapX.

In conclusion, Mig1 participates in many mitochondrial functions, such as cellular respiration, ROS tolerance, iron homeostasis, regulation of the iron-containing enzyme of the TCA cycle, and heme biosynthesis, as well as direct resistance to fluconazole. These studies support future efforts to target these mitochondrial functions to identify antifungal agents that may act synergistically with fluconazole to eliminate cryptococcal infections.

## MATERIALS AND METHODS

### Strains and media.

*C. neoformans* serotype A strain H99 and derivative mutants were routinely grown on YPD (1% yeast extract, 2% Bacto peptone, 2% dextrose, and 2% agar). Defined low-iron medium (LIM) was employed as described previously (56). Iron-replete medium was prepared by adding 100 µM ferric chloride (FeCl_3_) to LIM. YES medium was prepared as described previously (57). For growth assays on solid media, 10-fold serial dilutions of cells were spotted onto plates and incubated at 30°C or 37°C for 2 to 3 days before being photographed. The concentrations of each compound were as follows: 50 µg ⋅ ml^−1^ rotenone, 1 mM malonic acid, 10 mM salicylhydroxamic acid (SHAM), 2 µg ⋅ ml^−1^ antimycin A, 5 mM potassium cyanide (KCN), 0.05% H_2_O_2_, 50 µM plumbagin, 5 µg ⋅ ml^−1^ menadione, 500 µM paraquat, 50-µM diphenyleneiodonium chloride (DPI), 600 µM cobalt-chloride (CoCl_2_), 0.125% SDS, 1.5 M sodium chloride (NaCl), 1.5 M potassium chloride (KCl), 100 mM lithium chloride (LiCl), 10 µg ⋅ ml^−1^ sirolimus, 0.5 mg ⋅ ml^−1^ caffeine, 10 µg ⋅ ml^−1^ fluconazole, and 100 µg ⋅ ml^−1^ tetracycline.

### Construction of deletion mutants.

The *mig1*Δ::*NAT* and *hapX*Δ::*NAT* mutants were previously described (16, 17). The *mig1*Δ::*NEO* mutant and the *MIG1*::*NEO* complemented strain (subsequently referred as the *mig1*Δ::*MIG1* strain) were constructed by homologous recombination using the neomycin cassette and a three-step overlapping PCR method with the primers listed in Table S1 of the supplemental material (58). The resistance marker for neomycin (NEO) was amplified by PCR from plasmid pJAF1. In general, the gene-specific knockout primers 1 and 2 and 5 and 6 were used to amplify the flanking sequences of *C. neoformans* MIG1, and primers 3 and 4 were used to amplify the gene-specific deletion construct containing the resistance marker. The *mig1*::*NEO* construct was introduced into the H99 wild-type (WT) strain and the *hapX*Δ::*NAT* mutant of strain H99, and the *MIG1*::*NEO* construct for complementation was introduced into the *mig1*Δ::*NAT* mutant by biolistic transformation, as described previously (59). Gene deletion and reinsertion were verified by Southern hybridization (see Fig. S1 in the supplemental material).

10.1128/mSphere.00080-15.1 Table S1  Primers used in this study. Download Table S1, PDF file, 0.2 MB. Copyright © 2016 Caza et al. 2016 Caza et al. This content is distributed under the terms of the Creative Commons Attribution 4.0 International license.

10.1128/mSphere.00080-15.2 Table S2  Categories of differentially expressed genes. Download Table S2, PDF file, 0.7 MB. Copyright © 2016 Caza et al. 2016 Caza et al. This content is distributed under the terms of the Creative Commons Attribution 4.0 International license.

10.1128/mSphere.00080-15.3 Figure S1  (A) Southern blot of *mig1*Δ::*NEO* and *hapX*Δ*mig1*Δ mutants, the *mig1*Δ::*NAT* mutant, and the complemented *mig1*Δ::*MIG1* strain constructed in this study. (B) Genomic DNA of each mutant was extracted using standard protocols and, following RNase treatment, was digested with AvrII and Ssp1 prior to gel electrophoresis. Overnight transfer of the DNA to nitrocellulose membranes (Sigma) was performed using standard Southern blot methods, and ^32^P probes specific to the *MIG1* region were hybridized to the DNA on the membrane. Membranes were exposed to a phosphor screen (Amersham), and detection was performed using Pharos FX Molecular Imager software (Bio-Rad). Download Figure S1, TIF file, 0.1 MB. Copyright © 2016 Caza et al. 2016 Caza et al. This content is distributed under the terms of the Creative Commons Attribution 4.0 International license.

10.1128/mSphere.00080-15.4 Figure S2  Mig1 influence on the transcript levels of genes involved in the TCA cycle, amino acid biosynthesis, heme biosynthesis, and iron uptake. Transcript levels were assessed in a *mig1*Δ mutant compared to those of the WT for cells grown in LIM and in LIM plus 100 µM FeCl_3_. Transcript levels are shown for the putative mitochondrial aconitases *ACO1* (CNAG_01137), *ACO2* (CNAG_07908), *ACO3* (CNAG_02565), and *ACO4* (CNAG_03427), involved in the TCA cycle (A); *LEU1* (CNAG_00237), involved in leucine biosynthesis (B); *HEM3* (CNAG_01721) and *HEM4* (CNAG_01908), involved in heme biosynthesis (C); and *SIT6* (CNAG_07751) and *CIG1* (CNAG_ 01653), involved in iron and heme uptake (D). The experiments were carried out at least twice in triplicate. Values are reported as means ± SEM. Statistical significance was calculated using the unpaired two-tailed *t* test (*, *P* < 0.05). Download Figure S2, TIF file, 0.1 MB. Copyright © 2016 Caza et al. 2016 Caza et al. This content is distributed under the terms of the Creative Commons Attribution 4.0 International license.

The coding region of the candidate *MIG1/CREA* gene in *C. neoformans* (CNAG_06327) is 4,081 bp in length with 2 introns and encodes a polypeptide of 734 amino acids. A variant of *MIG1/CREA* is also described in the Broad Institute’s database (http://www.broadinstitute.org), with a sequence difference in the 5′ noncoding region. A phylogenetic tree for *MIG1* was constructed using the software MEGA 6, available at http://www.megasoftware.net/, and ortholog sequences were retrieved from the NCBI (http://www.ncbi.nlm.nih.gov/protein). An unrooted tree was created using the neighbor-joining consensus trees based on the calculated distances using 1,000 bootstrap replications. BLAST analysis was performed using NCBI Blast2Seq, and for the conserved domain alignment, we employed the ClustalW multiple-alignment method from the BioEdit software.

### RNA extraction and microarray experiments.

Gene expression in the WT strain and the *mig1*Δ::*NAT* mutant were determined by microarray analysis. Three biological replicates for each strain were grown in 10 ml of YPD overnight at 30°C. Cells were washed three times with low-iron water and counted. Cells were inoculated in 5 ml of LIM or LIM plus 100 µM FeCl_3_ at 5 × 10^7^ cells ⋅ ml^−1^, grown at 30°C for an additional 6 h, and harvested for RNA extraction. Cells were broken by bead beating using a Retsch MM301 mixer mill, and RNA was extracted with an RNeasy kit (Qiagen) and treated with DNase (Qiagen) according to the manufacturer’s recommendations. The quality of RNA was analyzed with an Agilent 2100 bioanalyzer, and cDNA was synthesized using a Verso cDNA kit (Thermo Scientific). The cDNAs were labeled with Cy3 or Cy5 for hybridization. The microarray experiments employed the publicly available whole-genome array of 70-mer oligonucleotides corresponding to each predicted gene in the *C. neoformans* genome (http://www.broadinstitute.org/annotation/genome/cryptococcus_neoformans/MultiHome.html). Hybridizations, data acquisition, and analysis were performed at the Duke Microarray Facility (http://genome.duke.edu/cores-and-services/sequencing-and-genomic-technologies). 

### RNA extraction and quantitative real-time RT-PCR.

The WT strain and the *mig1*Δ::*NEO* mutant were used for quantitative real-time RT-PCR. Cultures for three biological replicates of each strain were grown in 5 ml of YPD overnight at 30°C. Cells were washed three times with low-iron water and counted. Cells were transferred to 5 ml of LIM or LIM plus 100 µM FeCl_3_ at a density of 5 × 10^7^ cells ⋅ ml^−1^ and grown at 30°C for an additional 6 h. Cells were harvested and frozen in liquid nitrogen and stored at −80°C. Cell pellets were lysed by bead beating using a Retsch MM301 mixer mill; total RNA was extracted with an RNeasy kit (Qiagen) and treated with Turbo DNase (Ambion) according to the manufacturer’s recommendations. cDNA was synthesized using a high-capacity cDNA reverse transcription kit (Applied Biosystems) and oligo(dT) (Invitrogen). Primers for real-time RT-PCR were designed using Primer Express 3.0 (Applied Biosystems) and are listed in Table S1 of the supplemental material. Quantitative RT-PCR (qRT-PCR) was performed using Green-2-Go qPCR Mastermix (Bio Basic Inc.) and an Applied Biosystems 7500 Fast real-time PCR system. Relative gene expression was quantified using the 2^−ΔΔ*CT*^ method (where *CT* is the threshold cycle) with 18S rRNA as an endogenous control (60). Statistical significance was evaluated using the unpaired *t* test (GraphPad Prism 6 for Windows; GraphPad, San Diego, CA).

### Macrophage assays.

Macrophage infections were performed as follows. Briefly, macrophage-like J774.A1 cells were grown to 80% confluence in DMEM supplemented with 10% fetal bovine serum and 2 mM l-glutamine at 37°C with 5% CO_2_. Macrophages were stimulated 2 h prior to infection with 150 ng ⋅ ml^−1^ phorbol myristate acetate (PMA). Fungal cells were grown in YPD overnight, and PBS-washed cells were opsonized in DMEM with 0.5 µg ⋅ ml^−1^ of the monoclonal antibody 18B7 for 30 min at 37°C. Stimulated macrophages were infected with 2 × 10^5^ opsonized fungal cells (multiplicity of infection, 1:1) for 2 and 24 h at 37°C with 5% CO_2_. Macrophages containing internalized cryptococcal cells were washed thoroughly 3 times with PBS and then lysed with sterile water for 30 min at room temperature. Lysate dilutions were plated on YPD agar and incubated at 30°C for 48 h, at which time the resulting CFU were counted. Statistical significance of intracellular survival was determined by two-tailed unpaired *t* tests (GraphPad Prism 6 for Windows; GraphPad, San Diego, CA).

### Virulence assays in mice.

The virulence of the WT strain and of the *mig1*Δ::*NEO*, *hapX*Δ*::NAT*, and two *hapX*Δ *mig1*Δ mutants (designated A and B) was assessed using female BALB/c mice (4 to 6 weeks old) from Charles River Laboratories (Ontario, Canada). Two separate experiments were performed to assess the contribution of Mig1 and HapX for the virulence of *C. neoformans*. First, a mouse survival assay with assessment of fungal burden at the humane endpoint was executed. Second, fungal burden was assessed at 15 days postinfection for all mutants and the WT. In both experiments, fungal cells were grown in 5 ml of YPD at 30°C and washed twice with PBS (Invitrogen). Mice were anesthetized intraperitoneally with ketamine (80 mg ⋅ kg of body weight^−1^) and xylazine (5.5 mg ⋅ kg^−1^) and suspended on a silk thread by the superior incisors. A suspension of 5 × 10^4^ cells in 50 µl was slowly inoculated into the nares of the mice. The health status of the mice was monitored daily postinoculation (p.i.), and mice reaching the humane endpoint or at 15 days p.i. were euthanized by CO_2_ anoxia. Fungal burdens of organs (lungs, brain, liver, spleen, and kidney) and cardiac blood were assessed. The organs and blood were aseptically removed. Blood was retrieved from the heart using sterile syringes prerinsed with 500 units of heparin. Organs were homogenized in 1 ml of PBS using a Retsch MM301 mixer mill. The samples were serially diluted, plated on YPD containing chloramphenicol (30 µg ⋅ ml^−1^), and incubated at 30°C for 2 days; CFU were then counted. The protocol for the virulence assays (protocol A13-0093) was approved by the University of British Columbia Committee on Animal Care. Statistical analyses of survival differences in mice were performed with the log rank test, and a two-tailed unpaired Mann-Whitney test was used to assess the fungal load (GraphPad Prism 6 for Windows; GraphPad, San Diego, CA).

### Microarray data accession number.

The microarray data are available in the Gene Expression Omnibus database (http://www.ncbi.nlm.nih.gov/geo/) under accession number GSE76063.
