# Supplementary material for: The Zinc Finger Protein Mig1 Regulates Mitochondrial Function and Azole Drug Susceptibility in the Pathogenic Fungus Cryptococcus neoformans
Source: mSphere. 2016 Jan 13;1(1):e00080-15. doi: 10.1128/mSphere.00080-15 (PMC4863601; doi:10.1128/mSphere.00080-15)
Supplement: Table S1 [file sph001160060st1.pdf]

Table 1: Primer used in this study

**Primers used for mutagenesis**	
*mig1*Δ::*NEO* deletion construct	
MIG1-9PO	GGACAGCTCGCAAGTTCTTA
MIG1-Neo-2	CTGCAGATATCCATCACACTGGCGGCGATGGGATTTTGAGGG GAGGAGGATG
MIG1-Neo-3	CATCCTCCTCCCCTCAAAATCCCATCGCCGCCAGTGTGATGG ATATCTGCAG
MIG1-Neo-4	CTACGGATGTCCATCCAGCTCCATGCCGCCAGTGTGCTGGAA TTCGCCCTTGG
MIG1-Neo-5	ACCAAGGGCGAATTCCAGCACACTGGCGGCATGGAGCTGGA TGGACATCCGTAG
MIG1-6 R	ATCGGTCTCTACCCCTTCTGCAACAGCA
MIG1::NEO complementation construct	
MIG1-CP1	GAGAGGACAGCTCGCAAGTTCTTAAG
MIG1-CP2	GCAGATATCCATCACACTGGCGGCGTCCTTCTCATCAAATAC GACTCCTCTC
MIG1-CP3	GAGAGGAGTCGTATTTGATGAGAAGGACGCCGCCAGTGTGAT

	GGATATCTGC
MIG1-CP4	TGACAAGAGTAACGACGAGACATCTCTAAGTGTGCTGGAATT CGCCCTTGGT
MIG1-CP5	ACCAAGGGCGAATTCCAGCACACTTAGAGATGTCTCGTCGTT ACTCTTGTCA
MIG1-CP6	CTAGGTTGTCTCAGTAGTCGTTGTTAATG
**Primers used for qRT-PCR**	
QRT-MIG F	GTCCTCTATGTGATCGGGCGTTCTACAGGC
QRT-MIG R	TGGCATGTCTTGTCAATTCGTCGGAACGTG
HAPX-F1	TCGCACCTCTTCAGATGCTTT
HAPX-R1	CGAGAACCGCCACACAACT
Q1721-1F	TGCTGCCCATCCCGAAT
Q1721-2R	CCTGGCCCCTGAAAGGA
CIG1-RT-F	GGTGGTCCGGTTTCCTTCA
CIG1-RT-R	GACTCGTGGTCGTGCATAACA
Q1137-F	CCGTATCCACGAGACCAACT
Q1137-R	ATCATGTTGAGGGCAGAACC
Q7908-F	AAGGCTAGGGAAGAGCCTTG

Q7908-R	GCTTCTTGAGGTTGGTCTCG
Q3427-F	ACATCTGTTCACACCGACCA
Q3427-R	CAGGCTTCCAGAAAGCAATC
HEM4-F1	CGGGCCATACCTGTTCTTGT
HEM4-R1	GCGCGGTAGGCATCTCAT
Q2565-F	CGGTTCTTTCGGTGACATTT
Q2565-R	GTCTTCTCCCCTTCCTCACC
LEU1-F	CGGTATGCTTCCTCTTCAGC
LEU1-R	TCGGTGGACAAGGGTAAGAC
MIRB-F1	GCTCAGCGAGGTGTGCAAA
MIRB-R1	CGACATTCTCGACCAAGTCAAA
18S-RT-F	AACAGGTCTGTGATGCCCTTAGA
18S-RT-R	ACTCGCTGGCTCAGTCAGTGT
