# Supplementary material for: The Zinc Finger Protein Mig1 Regulates Mitochondrial Function and Azole Drug Susceptibility in the Pathogenic Fungus Cryptococcus neoformans
Source: mSphere. 2016 Jan 13;1(1):e00080-15. doi: 10.1128/mSphere.00080-15 (PMC4863601; doi:10.1128/mSphere.00080-15)
Supplement: Table S2 [file sph001160060st2.pdf]

Supplemental Table 2: Classified significant genes differently expressed in a *mig1*Δ mutant compared to the WT strain H99 (*mig1*Δ/WT) when grown in low iron media and high iron media.

CATEGORIES	DESCRIPTION	Fold change in low iron	Fold change in high iron	*P* value
**METABOLISM**				
**Nucleotides**				
CNAG_02294	Phosphoribosylaminoimidazole carboxylase (*ADE2*)	1.18		4.72E-08
CNAG_06694	Hydroxyisourate hydrolase (*URO2*)	1.18		2.09E-07
CNAG_02795	Phosphoribosylglycinamide formyltransferase	1.22		4.62E-06
CNAG_04192	Phosphoribosylformylglycinamidine synthase	1.23		1.68E-08
CNAG_06371	Guanine deaminase	1.21		2.88E-06
CNAG_03973	Hydantoinase	1.20		2.38E-07
CNAG_01915	Ribonucleoside-diphosphate reductase subunit M2	1.22		8.53E-09
CNAG_06708	dCMP deaminase		-1.09	3.29E-07
**Amino acids**				
CNAG_03701	3-phosphoshikimate 1-carboxyvinyltransferase	1.24		4.97E-07
CNAG_02270	Homoserine O-acetyltransferase (*MET2*)	1.24		6.72E-10
CNAG_04215	Sulfate adenylyltransferase (*MET3*)	1.27		7.83E-08
CNAG_03168	Sulfite reductase (NADPH) flavoprotein alpha-component	1.29		5.27E-07
CNAG_02202	Adenylyl-sulfate kinase homologue of *MET14*	1.13		8.35E-08
CNAG_04776	Homoserine dehydrogenase (*HOM6*)	1.14		2.54E-07
CNAG_01609	Cystathionine gamma-synthase	1.15		1.55E-06
CNAG_00237	3-isopropylmalate dehydratase (*LEU1*)	1.23		4.44E-12
CNAG_00743	Imidazoleglycerol phosphate synthase. cyclase subunit	1.14		6.95E-08
CNAG_06830	Histidinol dehydrogenase	1.16		5.71E-06
CNAG_01997	Prephenate dehydrogenase	1.09		2.70E-07
CNAG_01952	aryl-alcohol dehydrogenase	1.11		4.02E-06
CNAG_05070	Sulfite reductase (NADPH) hemoprotein. beta-component	1.36		1.10E-09
CNAG_01252	Iosulfate/3-mercaptopyruvate sulfurtransferase	1.16		2.17E-08

CNAG_05398	Phosphoserine transaminase	1.14		1.37E-06
CNAG_03588	L- aminoadipate-semialdehyde dehydrogenase (*LYS2*)	1.24		7.39E-08
CNAG_03720	D-tyrosyl-tRNA(Tyr) deacylase	1.16		1.62E-08
CNAG_03854	Lysine decarboxylase	-1.29		8.52E-11
CNAG_03812	Histidinol-phosphate transaminase		1.08	7.16E-06
CNAG_03280	Dihydrodipicolinate synthetase		1.27	1.19E-06
CNAG_05496	2-isopropylmalate synthase		1.16	4.04E-08
CNAG_07802	Class III aminotransferase		-1.14	1.78E-10
**Carbohydrates**				
CNAG_01776	2.4-dihydroxyhept-2-ene-1.7-dioic acid aldolase	1.15		2.08E-08
CNAG_06923	Xylulose 5-phosphate/fructose 6-phosphate phosphoketolase (*XFP2*)	1.22		4.79E-07
CNAG_05369	Beta-glucosidase	1.17		4.91E-07
CNAG_06659	Beta-hexosaminidase (*HEX1*)	1.14		4.25E-06
CNAG_04095	Alpha-L-rhamnosidase	1.13		2.42E-07
CNAG_04373	Alginate lyase	1.08		3.57E-09
CNAG_02552	Transketolase	-1.33		2.09E-06
CNAG_03096	UDP-glucose 4-epimerase	-1.17		8.11E-08
CNAG_03946	Galactokinase	-1.34		2.52E-11
CNAG_06260	Alpha-glucosidase involved in maltose catabolism	-1.31		2.71E-06
CNAG_04673	NAD dependent epimerase/dehydratase	-1.11		6.06E-06
CNAG_04122	Galactose dehydrogenase		1.15	2.09E-06
CNAG_06692	Glucosamine-phosphate N-acetyltransferase		-1.20	1.82E-08
CNAG_04523	Glyceraldehyde-3-phosphate dehydrogenase. type I		-1.24	1.23E-10
**Fatty acid**				
CNAG_02449	Long-chain fatty acid CoA ligase	1.09		3.34E-06
CNAG_01044	Dihydroceramidase	1.20		1.33E-07
CNAG_01116	Beta-ketoacyl reductase	1.09		4.16E-06
CNAG_02935	Malonic semialdehyde reductase	1.16		2.98E-08
CNAG_06783	Enoyl reductase	1.23		1.04E-10
CNAG_03623	Malonic semialdehyde reductase	-1.16		2.57E-06
CNAG_04652	Enoyl reductase	-1.25		1.21E-06
**Ergosterol**				
CNAG_01737	C-4 methyl sterol oxidase (*ERG25*)	1.15		2.60E-07
CNAG_02830	Delta24(24-1) sterol reductase (*ERG4*)	1.18		6.64E-06
CNAG_01771	Related to cytochrome P450 regulator Dap1	1.14		1.74E-07
**Folic acid**				
CNAG_02870	Folylpolyglutamate synthetase (*MET7*)	1.22		5.58E-06
CNAG_04010	GTP cyclohydrolase I (*FOL2*)	1.13		1.44E-07
CNAG_00760	Methylenetetrahydrofolate reductase (NADPH)	1.17		3.60E-07
CNAG_07796	Uroporphyrin-III C-methyltransferase	1.19		2.71E-07

**Co-enzyme A**				
CNAG_03738	Pantetheine-phosphate adenylyltransferase	1.15		4.00E-07
**Biotin**				
CNAG_00236	8-Amino-7-oxononanoate synthase	1.23		9.61E-08
**Heme**				
CNAG_01721	Porphobilinogen deaminase (*HEM3*)	1.08		3.04E-06
CNAG_02460	Coproporphyrinogen-III oxidase (*HEM13*)	1.20		2.83E-11
**Flavonoid**				
CNAG_03340	Flavonol synthase	1.18		1.49E-06
CNAG_02181	Dihydrokaempferol 4-reductase	-1.16		5.13E-07
**<u>TRANSPORT</u>**				
**Amino acid transporters**				
CNAG_02539	AAT family amino acid transporter	1.11		1.57E-06
CNAG_01074	Neutral amino acid transporter	1.13		2.14E-07
CNAG_00597	Amino acid transporter (*DIP5*)	-1.48		5.61E-09
CNAG_03988	Solute carrier family 38. member 11		-1.14	3.12E-07
**Carbohydrate transporters**				
CNAG_02479	Monosaccharide transporter	1.19		5.86E-09
CNAG_01683	Monosaccharide transporter (*STL1*)	-1.31		7.79E-08
CNAG_04210	Sugar transporter. probable maltose permease (*MALP*)	-1.47		2.14E-08
CNAG_06290	high-affinity glucose transporter (*SNF3*)	-1.29		2.82E-06
CNAG_03426	GDP-mannose transporter (*GMT2*)		1.25	1.12E-06
CNAG_02586	Sugar transporter		-1.16	1.74E-06
**Organic compound transporters**				
CNAG_05851	Glycerol transporter	1.13		2.21E-07
CNAG_04794	Spermine transporter	1.11		6.03E-06
CNAG_04818	Related to monocarboxylate transporter	1.19		3.79E-08
CNAG_04038	MFS quinate transporter (*QUTD*)	-1.17		3.29E-06
CNAG_04142	Tartrate transporter	-1.32		1.10E-07
CNAG_03051	Polyamine transporter	-1.25		8.46E-08
CNAG_05993	Transmembrane transporter (*LIZ1*)	-1.32		8.63E-09
CNAG_05667	Myo-inositol transporter (*ITR3B*)		-1.11	4.61E-06
CNAG_04536	Nicotinamide mononucleotide permease		-1.38	1.16E-09
**Ion transporters**				
CNAG_02221	Zinc ion transporter (*ZIP3*)	1.08		8.99E-07
CNAG_02806	Solute carrier family 30 (zinc transporter). member 1	1.16		1.01E-06
CNAG_05135	Calcium-transporting ATPase (*PMR1*)	1.14		6.90E-06
CNAG_01318	Sodium / hydrogen exhanger 3	1.15		4.47E-08
CNAG_02856	Potassium transport protein	1.26		9.31E-10

CNAG_02440	Cation-transporting ATPase	-1.35		2.12E-08
CNAG_04337	Cation diffusion facilitator		-1.10	1.44E-06
**Nuclear transport proteins**				
CNAG_04149	Nuclear pore complex protein Nup107	1.12		2.85E-06
CNAG_02203	Karyopherin/importin	1.25		5.66E-09
CNAG_06545	Pim1 protein + RNA transporter 2	1.15		7.63E-08
**Intracellular trafficking**				
CNAG_04684	Putative GTPase-activating protein (*SEC23*)	1.09		4.50E-06
CNAG_01720	Escrt-I complex protein (*VPS23*)	1.16		1.56E-06
CNAG_00582	Vacuolar transporter chaperone 1 (*VTC1*)	1.13		1.91E-07
CNAG_01263	Vacuolar transporter chaperone 4 (*VTC4*)	1.11		4.94E-06
CNAG_01759	Vacuolar membrane transport protein	1.20		1.00E-08
CNAG_01552	Blocked early in transport 1 (*BET1*)	1.21		1.10E-07
CNAG_00469	Endoplasmic reticulum protein may be involved in COP II vesicle transport	1.12		3.13E-07
**Other transporters**				
CNAG_00796	Putative ABC multidrug resistance transporter with similarity to Ste6 (*MDR1*)	1.16		1.22E-06
CNAG_00749	Alternative sulfate transporter	1.45		4.64E-09
CNAG_03524	Uncharacterized transmembrane receptor	1.29		9.31E-10
CNAG_03909	Putative transporter	1.17		3.36E-10
CNAG_01119	POT family proton-dependent oligopeptide transporter	-1.29		3.34E-06
CNAG_00905	MFS transporter	-1.46		1.40E-07
CNAG_01690	MFS transporter	-1.47		4.41E-11
CNAG_01960	Efflux protein EncT	-1.23		3.39E-07
CNAG_06758	Efflux protein	-1.42		9.66E-11
**CELL SIGNALLING**				
CNAG_00179	Heterotrimeric G-protein coupled receptor alpha subunit (*GPA2*)	1.24		5.19E-07
CNAG_02090	Heterotrimeric G-protein coupled receptor alpha subunit (*GPA3*)	1.27		5.61E-07
CNAG_01909	Signal transducer	1.21		5.82E-07
CNAG_03024	AGC protein kinase	1.16		5.18E-06
CNAG_06301	AGC family protein kinase (*SCH9*)	1.17		2.18E-07
CNAG_03202	Adenylate cyclase (*CAC1*)	1.21		1.37E-10
CNAG_04162	Protein kinase A catalytic subunit (*PKA2*)	1.16		1.13E-06
CNAG_05155	Protein tyrosine phosphatase (*PTP2*)	1.16		3.23E-06
CNAG_02446	Response regulator receiver protein	1.16		7.29E-08
CNAG_03500	Osmosensor	1.25		5.54E-09
CNAG_04574	Dolichyl-phosphate mannosyltransferase polypeptide	1.13		8.15E-07

	2 (*DMP2*)			
CNAG_01889	Glutathione S-transferase	-1.17		4.33E-06
CNAG_05998	Rho-GTPase (*RAC2*)	-1.18		1.46E-06
CNAG_00752	Transducin family protein	-1.36		2.97E-06
**<u>CELLULAR DIVISION</u>**				
**DNA replication**				
CNAG_03203	DNA polymerase epsilon p12 subunit	1.09		3.30E-06
CNAG_04703	DNA clamp loader	1.15		3.04E-09
CNAG_07686	Topoisomerase 1-associated factor 1	1.14		3.83E-06
CNAG_00665	DNA topoisomerase 2-associated protein PAT1	1.21		9.71E-07
CNAG_06744	Chromatin structure-remodeling complex subunit RSC9	1.11		3.05E-06
CNAG_05096	Histone deacetylase 1/2	1.20		1.29E-07
CNAG_05276	Histone deacetylase 1/2		1.08	2.34E-07
CNAG_03956	Replication factor C subunit 3/5		-1.11	1.04E-06
**DNA repair**				
CNAG_00076	A/G-specific adenine glycosylase	1.18		5.21E-07
CNAG_05201	DNA mismatch repair protein MSH4	1.13		1.06E-06
CNAG_00572	DNA excision repair protein ERCC-2	1.10		2.71E-06
CNAG_00445	nonhistone chromosomal protein (*NHP6B01*)	1.24		3.63E-09
CNAG_00395	Structure-specific endonuclease subunit SLX1	-1.14		2.54E-06
CNAG_01163	DNA repair and recombination protein (*RAD54*)	-1.24		5.86E-07
CNAG_02115	nonhistone chromosomal protein (*NHP6B02*)		1.10	1.62E-06
CNAG_03213	UV DNA damage endonuclease (*UVE1*)		1.23	8.50E-08
**Cell cycle**				
CNAG_01023	Cohesin complex subunit SCC1	1.32		1.70E-10
CNAG_03767	Cohesin complex subunit PSM1	1.20		2.26E-08
CNAG_01959	Condensin complex subunit 1	1.17		1.39E-06
CNAG_04648	Sister chromatid cohesion protein PDS5	1.17		7.74E-08
CNAG_03148	Nuclear condensin complex protein	1.18		2.45E-06
CNAG_05752	Kinesin microtuble motor protein (*KAR3*)	1.16		1.17E-08
CNAG_03453	kinesin-like protein (*KIP1*)	1.20		1.77E-08
CNAG_02721	Leucine repeat containing protein	1.24		6.07E-07
CNAG_00320	Anaphase-promoting complex subunit 3	1.20		3.67E-06
CNAG_07909	Meiotic recombinase Dmc1	1.15		5.16E-08
CNAG_02095	Cyclin	1.25		5.26E-09
CNAG_01572	Tyrosine phosphatase	1.31		1.20E-10
CNAG_00683	CMGC/CLK protein kinase	1.21		1.20E-06
CNAG_02051	Kinesin family member 20/23		1.15	8.17E-08
CNAG_05781	Dynein light chain LC8-type		-1.15	2.03E-07

CNAG_02186	Myosin regulatory light chain		-1.11	7.90E-07
**Mating**				
CNAG_03938	Nonmating-type specific pheromone G-protein coupled receptor (*CPR2*)	1.13		3.29E-11
CNAG_01234	Spore wall assembly-associated protein	1.23		2.30E-09
CNAG_06808	a-factor pheromone receptor (*STE3alpha*)	-1.12		1.02E-08
**GENE EXPRESSION**				
**Ribosome**				
CNAG_03762	AdoMet-dependent rRNA methyltransferase SPB1	1.13		5.82E-06
CNAG_01455	Large subunit ribosomal protein L39 (*RPL39alpha*)	1.13		1.28E-07
CNAG_01590	Large subunit ribosomal protein L32	1.19		2.33E-06
CNAG_03693	Large subunit ribosomal protein L11	1.15		4.25E-06
CNAG_05764	Small subunit ribosomal protein S5	1.19		3.18E-07
CNAG_05976	Nucleolar protein 58	-1.36		4.58E-06
CNAG_01791	Ribosome production factor 1	-1.27		3.48E-06
**Transcription**				
CNAG_02166	DNA-directed RNA polymerase II subunit RPB1	1.23		8.74E-09
CNAG_00068	Specific RNA polymerase II transcription factor	1.18		5.79E-06
CNAG_06754	RNA polymerase-associated protein (*CTR9*)	1.21		5.58E-09
CNAG_03940	Elongator complex protein 2	1.23		2.85E-07
CNAG_03603	ATP-dependent RNA helicase (*HAS1*)	1.08		4.75E-07
CNAG_05360	ATP-dependent RNA helicase (*MAK5*)	1.10		1.64E-06
CNAG_03205	ATP-dependent RNA helicase (*ROK1*)	1.13		7.10E-07
CNAG_06670	HIV Tat-specific factor 1	1.11		9.72E-08
CNAG_05830	Cytoplasmic protein with putative role in transcription	1.18		6.67E-07
CNAG_05782	Mortality factor 4-like protein 1	1.22		2.58E-06
CNAG_00347	DEAH box polypeptide 36 (RNA helicase)	-1.33		5.85E-08
CNAG_06322	Histone acetyltransferase (*SAS3*)		1.10	1.24E-06
CNAG_06706	Proline-. Glutamic acid- and Leucine-rich protein 1		-1.12	1.37E-06
**Transcription factors**				
CNAG_00156	Zinc finger transcription factor (*CRZ1*)	1.18		3.86E-07
CNAG_07435	CCAAT-binding transcription factor (*HAP2*)	1.29		5.39E-09
CNAG_04090	Activating transcription factor (*ATF1*)	1.24		5.22E-08
CNAG_03902	Transcriptional regulatory protein (*RDS2*)	1.20		9.36E-10
CNAG_00883	Zn2-Cys6 zinc-finger transcription factor (*ECM2201*)	1.15		6.98E-08
CNAG_01626	Predicted saga histone acetyltransferase complex component (*ADA2*)	1.21		1.20E-08
CNAG_05181	White-collar transcription factor; blue-light photoresponsive gene (*BWC1*)	1.10		5.60E-08

CNAG_04774	Nuclear protein	1.14		2.98E-06
CNAG_04637	Multiprotein bridging factor (*MBF1*)	-1.15		1.25E-08
CNAG_05785	Putative transcription factor (*STB4*)		-1.20	4.72E-06
**RNA processing**				
CNAG_00086	N2.N2-dimethylguanosine tRNA methyltransferase	1.13		5.17E-09
CNAG_05101	U3 small nucleolar RNA-associated protein 12	1.14		9.78E-07
CNAG_06798	U3 small nucleolar RNA-associated protein 18	1.10		4.31E-07
CNAG_01146	U3 small nucleolar RNA-associated protein 22	1.19		4.02E-06
CNAG_03491	Cleavage and polyadenylation specificity factor subunit 5	1.31		3.86E-07
CNAG_01526	Cleavage and polyadenylation specificity factor subunit 4	1.15		6.45E-07
CNAG_04821	PAB-dependent poly(A)-specific ribonuclease subunit PAN3	1.23		5.37E-08
CNAG_02028	CMGC/SRPK protein kinase	1.18		1.39E-11
CNAG_05457	Pentatricopeptide repeat protein	1.12		5.87E-06
CNAG_00635	RNA binding protein	1.17		3.47E-06
CNAG_04028	RNA binding protein	-1.21		6.88E-07
**Spliceosome**				
CNAG_01809	U4/U6 small nuclear ribonucleoprotein PRP3	1.16		2.03E-06
CNAG_01091	U5 small nuclear ribonucleoprotein component	1.17		3.14E-07
CNAG_05848	Splicing factor U2AF 65 kDa subunit	1.21		4.04E-06
**RNA degradation**				
CNAG_04609	Argonaute protein (*AGO1*)	1.20		2.59E-09
CNAG_02745	Dicer (*DCR2*)	1.18		1.57E-06
CNAG_03314	YTH domain family 2	1.20		1.93E-06
**Translation**				
CNAG_03606	Translation initiation factor aIF-2	1.14		8.07E-07
CNAG_02507	Translation initiation factor 3 subunit H	1.14		1.15E-06
CNAG_00238	Isoleucine-tRNA ligase	1.16		9.25E-07
CNAG_04800	Phenylalanine-tRNA ligase. alpha subunit	1.13		3.72E-08
CNAG_06755	Threonine-tRNA ligase	1.21		2.19E-07
CNAG_03792	tRNA adenylyltransferase	1.11		1.11E-06
CNAG_00292	Zinc finger protein (*ZRP1*)	-1.37		4.74E-08
**<u>PROTEINS PROCESSING</u>**				
**Post-translational modification**				
CNAG_01592	Protein-S-isoprenylcysteine O-methyltransferase	1.20		5.80E-10
CNAG_07780	Geranylgeranyl diphosphate synthase	-1.18		1.59E-06
CNAG_01520	Histone-arginine methyltransferase CARM1	-1.32		3.05E-08
CNAG_06221	Peptidyl-diphthamide biosynthesis protein 2	-1.15		7.17E-06
**Protein folding**				

CNAG_02500	Calnexin	-1.26		1.28E-06
CNAG_00447	T-complex protein 1 subunit beta	-1.30		1.67E-06
CNAG_04304	T-complex protein 1 subunit zeta	-1.11		9.26E-07
CNAG_06240	Protein disulfide-isomerase	-1.22		2.28E-06
**STRUCTURE**				
**Membrane**				
CNAG_02415	Annexin XIV	1.18		4.69E-09
CNAG_02268	Nuclear membrane protein	1.09		1.72E-06
CNAG_04453	Inositol-phosphoryl ceramide synthase (*IPC1*)	1.17		5.79E-06
CNAG_06543	Ethanolaminephosphotransferase	1.06		6.94E-06
CNAG_00388	1-phosphatidylinositol-4-phosphate 5-kinase	1.15		6.43E-06
CNAG_05083	Esterase/lipase	1.11		3.78E-09
CNAG_07544	Sterol esterase	1.09		8.95E-09
CNAG_05266	Membrane protein	-1.20		5.88E-09
CNAG_00923	Membrane protein	-1.15		7.23E-08
CNAG_03991	Integral membrane protein		1.34	1.29E-11
CNAG_01711	Glycerol-3-phosphate O-acyltransferase		1.05	1.37E-06
**Cell wall**				
CNAG_03234	Cell wall surface anchor protein	1.48		9.81E-10
CNAG_02871	GPI mannosyltransferase 2	1.09		2.48E-06
CNAG_05080	Calcofluor white hypersensitive protein	1.16		6.55E-07
CNAG_06487	Putative chitin synthase (*CHS6*)	1.14		1.30E-07
CNAG_06678	Putative chitin synthase regulator (*CSR1*)	1.15		5.60E-06
CNAG_07499	Putative chitin synthase (*CHS8*)	1.12		9.00E-09
CNAG_05818	Putative chitin synthase (*CHS5*)	-1.15		2.19E-06
CNAG_04245	Endochitinase (*CHI22*)	1.31		2.17E-10
**Capsule**				
CNAG_00746	Capsular associated protein (*CAS35*)	1.12		2.90E-08
CNAG_00721	Alpha-1.3-mannosyltransferase (*CAP59*)	1.21		2.92E-06
CNAG_02885	Capsule-associated protein (*CAP64*)	1.12		6.11E-07
CNAG_07554	Capsule-associated protein (*CAP10*)	1.18		2.62E-07
CNAG_01341	Mannose-6-phosphate isomerase (*MAN1*)		-1.13	3.50E-06
**STRESS RESPONSE**				
**Chaperon & Heat shock proteins**				
CNAG_01696	Chaperone DnaJ	-1.52		8.28E-12
CNAG_00305	Chaperone activator	-1.42		3.59E-09
CNAG_03944	Chaperone regulator	-1.40		3.52E-09
CNAG_01185	Hsp70-like protein	-1.47		1.02E-09
CNAG_01347	Co-chaperone	-1.14		1.77E-07

CNAG_06443	Putative ATPase and ER protein chaperone similar to Kar2 (*SSA1*)	-1.59		3.20E-08
CNAG_06106	Chaperone regulator	-1.57		2.88E-09
CNAG_00287	Cytoplasmic protein putative Hsc70 co-chaperone	-1.34		1.41E-09
**Iron homeostasis**				
CNAG_06524	Ferric reductase (*FRE3*)	1.09		4.92E-06
CNAG_04202	Cytosolic Fe-S cluster assembly factor (*NAR1*)	1.14		4.62E-06
CNAG_02959	Iron permease (*CFT2*)	1.21		1.77E-10
CNAG_04314	NAD+ kinase (associated with ferric reductase activity)	1.14		4.46E-06
CNAG_03465	Laccase 1 (*LAC1*)	-1.36		1.59E-06
CNAG_03464	Laccase 2 (*LAC2*)	-1.23		4.10E-07
CNAG_06242	Major iron permease (*CFT1*)		-1.25	5.31E-09
**Proteasome**				
CNAG_00062	26S proteasome regulatory subunit N3	1.24		7.65E-07
CNAG_02395	Ubiquitin carboxyl-terminal hydrolase 9/13	1.19		1.46E-09
CNAG_00426	UBA/TS-N domain-containing protein	1.29		6.02E-06
CNAG_02568	UBA/TS-N domain-containing protein	1.21		5.57E-06
CNAG_01732	Nuclear protein localization protein 4	1.15		1.18E-06
CNAG_03039	DNA damage-inducible protein 1	-1.21		3.41E-06
CNAG_04328	Ubiquitin-conjugating enzyme E2 I	-1.12		7.03E-07
CNAG_03598	Ubiquilin	-1.36		3.48E-07
CNAG_03816	20S proteasome subunit beta 1	-1.11		2.62E-06
CNAG_04071	20S proteasome subunit alpha 6	-1.16		3.99E-07
CNAG_02239	26S protease regulatory subunit 4	-1.42		4.13E-07
CNAG_00046	26S proteasome regulatory subunit N8	-1.31		7.58E-09
CNAG_01746	E3 ubiquitin-protein ligase RNF14	-1.10		9.30E-08
CNAG_01920	Ubiquitin precursor (*UBI4*)		-1.09	1.13E-06
CNAG_06402	26S proteasome complex subunit DSS1		-1.08	5.23E-06
CNAG_02214	Ubiquitin conjugating enzyme (*UBC6-2*)		-1.08	3.02E-06
**ENZYMES**				
**Oxidoreductases**				
CNAG_06081	Glucose oxidase	1.22		4.04E-06
CNAG_01540	Dehydrogenase	1.14		1.88E-06
CNAG_03865	Putative oxidoreductase	1.12		9.91E-07
CNAG_05114	Peroxisomal copper amine oxidase	1.17		4.41E-09
CNAG_01794	2-hydroxyacid dehydrogenase	1.13		1.83E-06
CNAG_05723	Alcohol dehydrogenase	1.10		1.53E-06
CNAG_00735	Aldehyde dehydrogenase family 7 member A1	1.17		4.31E-09
CNAG_04467	Succinate-semialdehyde dehydrogenase (NADP+)	1.11		6.83E-09

	(*UGA2*)			
CNAG_07626	NAD-binding Rossmann fold oxidoreductase	1.28		6.07E-13
CNAG_04657	Short chain dehydrogenase	1.25		4.21E-11
CNAG_05299	Oxidoreductase	-1.35		9.55E-07
CNAG_03176	Endoplasmic oxidoreductin 1	-1.42		3.55E-08
CNAG_05115	Sarcosine oxidase	-1.19		5.13E-06
CNAG_07491	Glutaredoxin	-1.12		4.85E-06
CNAG_04981	Catalase 1 similar to spore-specific catalases (*CAT1*)	-1.53		5.90E-06
CNAG_01605	Rossman fold oxidoreductase		1.08	4.13E-06
CNAG_00984	Glucose and ribitol dehydrogenase		1.24	3.80E-06
CNAG_03199	FAD dependent oxidoreductase		1.10	6.32E-06
CNAG_00542	Salicylate hydroxylase		-1.15	3.94E-07
CNAG_02553	Short-chain dehydrogenase		-1.20	1.94E-08
CNAG_01464	Flavohemoglobin (*FHB1*)		-1.16	6.36E-06
CNAG_01949	Chlorophyll synthesis pathway protein BchC		-1.13	1.88E-08
CNAG_06672	Formate dehydrogenase		-1.23	7.85E-10
**Transferases**				
CNAG_04860	Xylosylphosphotransferase (*XPT1*)	1.22		7.04E-06
CNAG_04195	O-methyltransferase	1.12		8.73E-07
**Hydrolases**				
CNAG_04869	Para-nitrobenzyl esterase (*PNB1*)	1.26		1.71E-06
CNAG_04104	Mucin-desulfating sulfatase	1.19		5.16E-11
CNAG_01744	Phosphatase	1.13		7.02E-07
CNAG_06601	Amidohydrolase	1.16		6.79E-06
CNAG_01072	Amidase	1.32		6.86E-08
CNAG_02980	Membrane dipeptidase	1.18		1.84E-08
CNAG_06640	Cathepsin A (carboxypeptidase C)	1.17		6.56E-06
CNAG_01879	Cathepsin A (carboxypeptidase C)	1.12		5.73E-07
CNAG_00919	Carboxypeptidase D	1.16		1.58E-06
CNAG_01266	ATP-dependent protease La	1.16		3.51E-06
CNAG_04269	Leucyl aminopeptidase	1.22		1.32E-10
CNAG_07520	Endopeptidase	1.20		3.62E-10
CNAG_00150	Peptidase	1.28		1.05E-07
CNAG_04619	Prolyl oligopeptidase	1.18		2.41E-06
CNAG_00098	Palmitoyl-protein thioesterase	1.22		2.23E-08
CNAG_04735	Metalloproteinase (*MEP1*)	-1.12		2.17E-06
CNAG_06482	Amidase	-1.15		1.97E-06
**OTHER**				
CNAG_02588	Avenacinase	1.14		5.10E-08
CNAG_07422	Delayed-type hypersensitivity antigen (*DHA1*)	1.17		8.77E-07

CNAG_04253	Transmembrane protein	1.17		7.13E-08
CNAG_04403	CCCH zinc finger protein	1.17		2.38E-06
CNAG_05707	Cytoplasmic protein	1.09		3.57E-08
CNAG_01015	Cytoplasmic protein	1.60		3.39E-09
CNAG_03621	Cyclophilin A (*CPA2*)	-1.21		9.13E-08
CNAG_06220	Allergen	-1.20		5.42E-13
CNAG_01763	Cytoplasmic protein	-1.19		1.27E-10
CNAG_06383	Cytoplasmic protein	-1.12		2.80E-06
CNAG_02473	Endoplasmic reticulum protein		-1.10	7.16E-07
CNAG_03012	Quorum sensing-like molecule (*CQS1*)		-1.31	4.53E-08
**<u>MITOCHONDRIA FUNCTIONS</u>**				
**<u>CELLULAR RESPIRATION</u>**				
**Oxidative decarboxylation of pyruvate**				
CNAG_03509	Pyruvate dehydrogenase protein X component	1.18		7.61E-09
CNAG_05059	Pyruvate dehydrogenase E1 component subunit beta	1.16		8.88E-07
CNAG_04108	Pyruvate dehydrogenase kinase	1.25		6.98E-06
**TCA Cycle**				
CNAG_05653	Malate synthase (*MLS1*)	1.19		1.10E-06
CNAG_00061	Citrate synthase (*CIT1*)	1.12		1.38E-06
CNAG_01137	Mitochondrial aconitase hydratase (*ACO1*)	1.22		4.01E-07
CNAG_03427	Aconitase hydratase (*ACO4*)	1.16		1.64E-06
**Respiratory chain proteins**				
CNAG_02792	Mitochondrial BCS1 respiratory chain	1.13		1.27E-07
CNAG_04529	Cytochrome c oxidase-assembly factor (*COX16*)	1.15		2.84E-07
CNAG_02609	3-demethylubiquinone-9 3-O-methyltransferase	1.14		3.52E-06
CNAG_04264	Ubiquinone biosynthesis monooxygenase (*COQ6*)	1.16		1.08E-09
CNAG_03618	Quinone reductase (NADPH2)	-1.22		5.19E-06
CNAG_00462	Electron-transferring-flavoprotein dehydrogenase		1.22	3.17E-09
**<u>TRANSPORT</u>**				
CNAG_01529	Mitochondrial phosphate transporter. member 23/24/25/41	1.24		1.87E-09
CNAG_05283	Mitochondrial carrier protein. member 16	1.13		1.59E-07
CNAG_02917	Mitochondrial solute carrier protein (*YHM1*)	1.17		1.45E-06
CNAG_01752	Mitochondrial 2-oxodicarboxylate transporter. member 21	1.14		1.27E-09
CNAG_03452	AFG1 family mitochondrial ATPase	1.20		1.89E-07
CNAG_03512	Putative mitochondrial K+/H+ exchanger (KHE) protein	1.17		4.24E-07
CNAG_05150	ATP-binding cassette (ABC) transporter. mitochondrial precursor	1.11		2.65E-08

CNAG_05300	Mitochondrial carrier protein	-1.23		4.36E-06
**<u>MTOCHONDRIAL PROTEINS</u>**				
CNAG_02131	Iron-sulfur cluster assembly protein (*ISA1*)	1.12		5.59E-06
CNAG_05194	Lipoyl acid synthase	1.20		2.15E-06
CNAG_06316	Glycine cleavage system H protein	1.09		2.89E-06
CNAG_01594	Glycine cleavage system P protein	1.40		5.39E-12
CNAG_02793	Kynurenine 3-monooxygenase	1.10		2.37E-06
CNAG_01846	Flavoprotein	-1.34		5.67E-06
CNAG_02976	Riboflavin kinase	-1.14		4.95E-06
CNAG_02488	Methionyl-tRNA formyltransferase	1.16		3.77E-06
CNAG_06446	Mitochondrial splicing suppressor	1.35		1.01E-09
CNAG_01906	Mitochondrial distribution and morphology protein 12	1.16		2.15E-08
CNAG_07810	Mitochondrion protein	1.22		1.75E-07
CNAG_03507	Mitochondrial processing peptidase beta subunit	1.21		4.38E-06
CNAG_04017	Protein-methionine-R-oxide reductase (*MXR2*)	-1.19		2.10E-07
CNAG_05932	Peptidyl-prolyl cis-trans isomerase D (Cyclophilin D)	-1.52		1.45E-09
CNAG_00371	Methylglutaconyl-CoA hydratase		-1.08	5.66E-07
CNAG_02049	Proline oxidase (*PUT1*)		-1.24	1.25E-07

P values were determined using an Adaptive Holm multiple testing correction.
