# Supplementary figures and images for: The Zinc Finger Protein Mig1 Regulates Mitochondrial Function and Azole Drug Susceptibility in the Pathogenic Fungus Cryptococcus neoformans
Source: mSphere. 2016 Jan 13;1(1):e00080-15. doi: 10.1128/mSphere.00080-15 (PMC4863601; doi:10.1128/mSphere.00080-15)

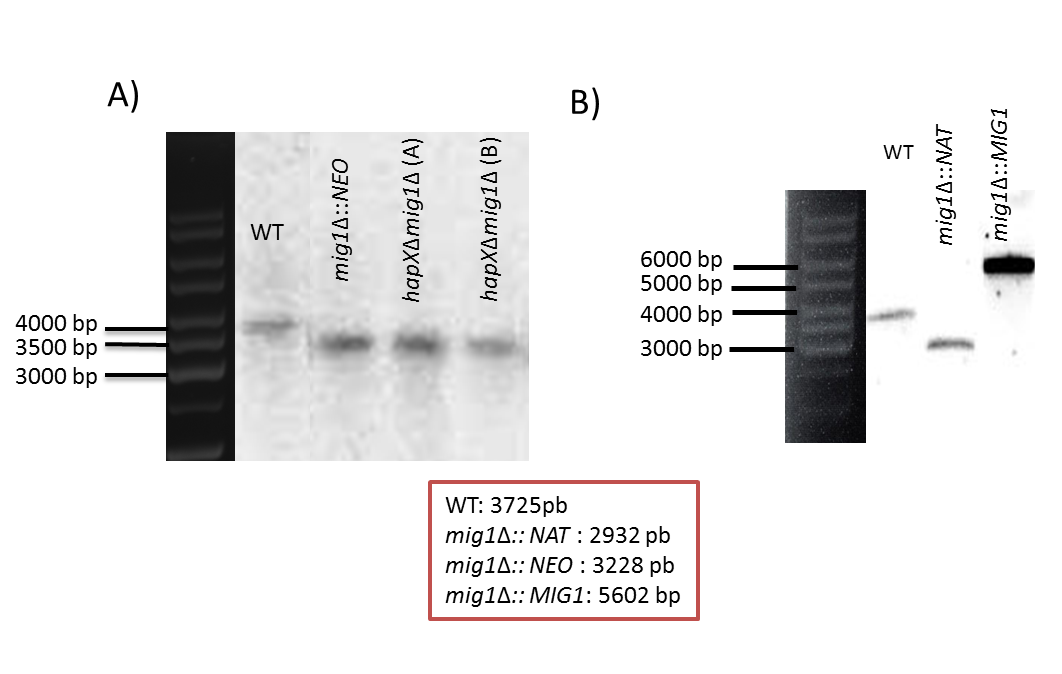

Supplement: Figure S1 [file sph001160060sf3.tif]
